# Basic stochastic model for tumor virotherapy

**DOI:** 10.3934/mbe.2020236

**Published:** 2020-06-17

**Authors:** Tuan Anh Phan, Jianjun Paul Tian

**Affiliations:** 1 Department of Mathematical Sciences, New Mexico State University, Las Cruces, New Mexico, 88001, USA

**Keywords:** virotherapy, viral burst size, Ito stochastic differential equation, ergodic invariant probability measure

## Abstract

The complexity of oncolytic virotherapy arises from many factors. In this study, we incorporate environmental noise and stochastic effects to our basic deterministic model and propose a stochastic model for viral therapy in terms of Ito stochastic differential equations. We conduct a detailed analysis of the model using boundary methods. We find two combined parameters, one describes possibilities of eradicating tumors and one is an increasing function of the viral burst size, which serve as thresholds to classify asymptotical dynamics of the model solution paths. We show there are three ergodic invariant probability measures which correspond to equilibrium states of the deterministic model, and extra possibility to eradicate tumor due to strong variance of tumor growth rate and medium viral burst size. Numerical analysis demonstrates several typical solution paths with biological explanations. In addition, we provide some medical interpretations and implications.

## 1. Introduction

Cancer is a genetic disease. It is caused by changes to genes, which control how cells grow and divide. A DNA change can cause genes involved in normal cells to become oncogenes. A oncogene is difficult to be turned off and so it causes cells grow without limits. When too many cells are accumulated, they form a solid tumor, which is masses of tissue. Cancer therapy is a broad area of research, which may have three subfields: immunotherapy, gene therapy, and oncolytic virotherapy. Immunotherapy relies on the concept of stimulating the body’s immune system to recognize and destroy cancer cells. Cancer cells harvested from patients are grown in vitro. Then these cells are engineered to be more recognizable to immune system by some substances or genes. These altered cells are grown in vitro and killed and their contents are incorporated into a vaccine that will be administered to patients, in order to boost the patients’ immune responses. But this method had limited success [1]. Gene therapy also is called gene transfer treatment which refers to the insertion of a foreign gene into the cancer cell or surrounding tissue to express specific genes such as suicide genes. This method does not rely on the immune system. Typically, replication-incompetent viruses, such as modified strains of adenovirus, have been used to deliver these genes. However, this technique met a lot of difficulties such as gene silence, the gene not being expressed for long enough time period [2].

In this paper, we will focus on mathematical analysis of the third type of cancer treatments, known as oncolytic virotherapy. Oncolytic viral therapy is considered to be a promising therapeutic strategy to treat solid tumors [3] and has shown its efficacy in clinical trials [4, 5]. This treatment involves the use of oncolytic viruses, namely genetically modified viruses to selectively infect cancer cells and induce cell death through lysis and further propagation of the virus. A number of viruses such as adenovirus, ONYX-15 and CV706, herpes simplex virus 1, and wild-type Newcastle disease virus have been used for such purposes. These viruses are shown to be unharmful to normal cells and tumor specific. In contrast to gene transfer treatments which utilize replication-incompetent viruses to alter the characteristics of cancer cells, oncolytic viruses have the ability to selectively replicate within the target cancel cell, resulting in the amplification effect in areas of tumor growth, allowing for safer doses of viral agent to be used in treatment [6].

Mathematical models formulated in terms of ordinary differential equations (ODEs) have been applied to understand spreading dynamics of oncolytic viruses through tumors for nearly twenty years. The early ODE model was proposed by Wodarz [7, 8], and was generalized by Dingli et al [9] later on. These models were formulated with three physical variables: uninfected tumor cells, a free virus population, and tumor cells infected by virus particles. The uninfected tumor cells were assumed to undergo logistic growth, and infected by virus particles, which multiply rapidly with infected tumor cells. Infected tumor cells were removed from the system due to natural or virus-inflicted death, resulting in new virus particles bursting to the free virus population. Motivated by experimental evidence, Bajzer et al [10] suggested that the forming of syncytia by fusing of uninfected and infected tumor cells rather than the free virus particles was the physical mechanism which drives intratumor virus spreading. Komarova and Wodarz [11] proposed and analyzed several general mathematical formulations for oncolytic virus infection in terms of systems of two ordinary differential equations, which categorized two types of virus spread, slow and fast spread. Our work [12] proposed a simple system of three ordinary differential equations to describe the interactions among uninfected tumor cells, infected tumor cells, and oncolytic viruses. Our analytic and numerical results concluded that the oncolytic viral dynamics is mainly determined by the viral burst size. To further understand the complexity of immune responses in virotherapy, we incorporated the innate immune response into our basic model for virotherapy and investigated how the innate immunity affects the outcome of virotherapy [13].

Stochastic effects are encountered in many biological and medical systems. Stochastic models may be able to capture some stochastic effects or variations in dynamics of biological and medical problems. In recent years, several attempts have been made to characterize viral dynamics for oncolytic virotherapy using stochastic differential equations (SDEs) such as Yuan and Allen [14], Kim et al [15], and Rajalakshmi et al [16, 17]. Most of these stochastic models were formulated by transforming ODE systems using the method proposed in [18]. These transformed SDE models may have some computational advantages. In this study, we propose a system of stochastic differential equations for tumor virotherapy and carry out its analysis and computation based on some suggestions from research presented in articles [19, 20, 21].

In [12], we proposed a common basic deterministic model for oncolytic virotherapy that includes the virus burst size *b* explicitly as follows,

(1.1) $$\frac{dx}{dt}=\rho x\left(1-\frac{x+y}{C}\right)-\beta xv,\;\frac{dy}{dt}=\beta xv-\delta y,\;\frac{dv}{dt}=b\delta y-\beta xv-\gamma v,$$

in which *x* stands for the uninfected tumor cell population, *y* the infected tumor cell population, and *v* the free virus population. The tumor growth is modeled by a logistic pattern with the growth rate *ρ* and carrying capacity of the tumor size *C*. The coefficient *β* represents the infectivity of the virus. The infected tumor cells die with a rate *δy*, which means the average life time of infected tumor cells is $\frac{1}{\delta }$. The viral burst size *b* is the number of new viruses released from a lysis of an infected tumor cell. The term *γv* is the clearance rate of free virus particles by various reasons including non-specific binding and generation of defective interfering particles.

There are several ways to incorporating environmental noise or stochastic effects into mathematical models. Suppose *P* is a population, its growth or change is modeled $\frac{dP}{dt}=f(t,P)$ in the deterministic situation. To count for environmental noise and stochastic effect, we may consider that each individual in the population make almost same contribution to the stochastic effects and receive the same environmental noise. Then, we may model the environmental noise and stochastic effects of the population is proportional to the population *P*. In other words, the environmental noise and stochastic effects can be represented by *τPξ*, where *ξ* is the unit noise [22] and *τ* can be thought as a way to measure an average variation of each individual. In general, we take the noise to be white noise $\xi =\frac{dW}{dt}$, where *W* = *W*(*t*) is the standard Wiener Process. So, we obtain a Ito stochastic differential equation $\frac{dP}{dt}=f(t,P)+\tau P\frac{dW}{dt}$, or *dP* = *f*(*t*, *P*)*dt* + *τPdW* as a stochastic model for the population *P*. We may call the noise added this way the linear noise. For our model (1.1), we will incorporate linear noise to the infected tumor cell population and free virus population. that is, we will add $\tau_{2}y\frac{dW_{2}}{dt}$ and $\tau_{3}v\frac{dW_{3}}{dt}$ to the second and the third equations of (1.1), respectively. However, for the uninfected tumor cell population, we incorporate environmental effects into per capital growth rate *ρ*. That is, we replace *ρ* by $\rho +\tau_{1}\frac{dW_{1}}{dt}$, where *τ*_1_ represent the strength of the noise contributed by each tumor cells. It should be assumed that *W*_1_, *W*_2_, and *W*_3_ are mutually independent Wiener processes. Such, we obtain a system of three Ito stochastic differential equations which is a basic stochastic model for oncolytic viral therapy as follows.


(1.2) $$dx=\left[\rho x\left(1-\frac{x+y}{C}\right)-\beta xv\right]dt+\tau_{1}x\left(1-\frac{x+y}{C}\right)dW_{1},\;dy=(\beta xv-\delta y)dt+\tau_{2}ydW_{2},\;dv=(b\delta y-\beta xv-\gamma v)dt+\tau_{3}vdW_{3}.$$

The analysis of the deterministic system (1.1) (see [12]) shows that the virus burst size *b* plays a crucial role in determining its dynamics. We found two important thresholds of the burst size that give a complete picture of dynamical behavior of (1.1). Our aim in this work is to analyze the SDE system (1.2) in order to find thresholds under which we can identify the extinction or persistence of the tumor cells and, furthermore, figure out how noise intensities affect the dynamics of the SDE system (1.2).

The rest of this paper is organized as follows. In Section 2, we simplify the stochastic system (1.2), introduce notations, present our results, state medical interpretations. In Section 3, we analyze our model using boundary analysis technique, and prove our results. In Section 4, by means of published data, we demonstrate typical dynamic behaviors of our stochastic model by numerical simulations and explain possible biological meanings. We also provide a brief discussion, some open problems, and possible future work. Finally, we present some basic properties of Generalized Inverse Gaussian distribution in Appendix.

## 2. Results and interpretations

First of all, for simplicity, we non-dimensionalize the system (1.2) by setting *T* = *δt*, $x=C\bar{x}$, $y=C\bar{y}$, $v=C\bar{v}$, $r=\frac{\rho }{\delta }$, $a=\frac{\beta C}{\delta }$, $c=\frac{\gamma }{\delta }$, $\tau_{1}={\bar{\tau }}_{1}$, $\tau_{2}={\bar{\tau }}_{2}$, and $\tau_{3}={\bar{\tau }}_{3}$. Then (1.2) becomes

(2.1) $$d\bar{x}=[r\bar{x}(1-\bar{x}-\bar{y})-a\bar{xv}]dT+{\bar{\tau }}_{1}\bar{x}(1-\bar{x}-\bar{y})dW_{1},\;d\bar{y}=(a\bar{xv}-\bar{y})dT+{\bar{\tau }}_{2}\bar{y}dW_{2},\;d\bar{v}=(b\bar{y}-a\bar{xv}-c\bar{v})dT+{\bar{\tau }}_{3}\bar{v}dW_{3}.$$

Dropping all bars over the parameters and variables and writing *T* as *t*, we obtain

(2.2) $$dx=[rx(1-x-y)-axv]dt+\tau_{1}x(1-x-y)dW_{1},\;dy=(axv-y)dt+\tau_{2}ydW_{2},\;dv=(by-axv-cv)dt+\tau_{3}vdW_{3}.$$

All parameters are positive. Assume that we are working on a complete probability space $\left(\Omega ,F,{\left\{F_{t}\right\}}_{t\geq 0},\mathbb{P}\right)$ with a filtration ${\left\{F_{t}\right\}}_{t\geq 0}$ satisfying the usual condition. The process given by the solution to the system (2.2) will be denoted by *u* or *u*(*t*) = (*x*(*t*), *y*(*t*), *v*(*t*)), *t* ≥ 0. We denote the drift term and the diffusion term of the system (2.2) by

$$f(u)=\left[\begin{array}{c} rx(1-x-y)-axv\\ axv-y\\ by-axv-cv \end{array}\right],\;\text{and}\;g(u)=\left[\begin{array}{ccc} \tau_{1}x(1-x-y) & 0 & 0\\ 0 & \tau_{2}y & 0\\ 0 & 0 & \tau_{3}v \end{array}\right].$$

Let *L* be the infinitesimal generator of the process *u* and, for any smooth enough functions $F:{\mathbb{R}}_{+}^{3}:={\left[0,\infty \right)}^{3}\to \mathbb{R}$, the generator *L* acts as

$$LF(u)=F_{u}\cdot f(u)+\frac{1}{2}\mathrm{trace}\left(g(u)g{\left(u\right)}^{T}F_{uu}\right),$$

where *F*_*u*_ is the gradient of *F* and *F*_*uu*_ is the Hessian matrix of *F*. We use ${\mathbb{P}}_{u}$ or ${\mathbb{P}}_{x,y,v}$ to denote the probability law on Ω when the solution path starts at *u* = (*x*, *y*, *v*) and $E_{u}$ or $E_{x,y,v}$ is the expectation corresponding to ${\mathbb{P}}_{u}$.

Based on the results (see Theorem 1.1 and Theorem 1.3 in [23]) about asymptotic behaviors of stochastic Kolmogorov systems in non-compact domains, we derive a sufficient and almost necessary condition to determine the extinction and persistence of populations of uninfected tumor cells, infected tumor cells, and free viruses. However, these results cannot be applied directly to our model because the drift term of the system (2.2) is not in Kolmogorov form as that of the system in [23]. To apply these results, we need to change variables. In view of (2.2), set the transformation of variables *x* = *x*, *y* = *y*, *v* = *yz*, or $z=\frac{v}{y}$, and use Ito’s formula, we get

$$dz=vd\left(\frac{1}{y}\right)+\frac{1}{y}dv+dvd\left(\frac{1}{y}\right)\;\;\;\;\;\;\;\;=v\left[\left(-ax\frac{v}{y^{2}}+\frac{1}{y}+\tau_{2}^{2}\frac{1}{y}\right)dt-\tau_{2}\frac{1}{y}dW_{2}\right]+\frac{1}{y}\left[(by-axv-cv)dt+\tau_{3}vdW_{3}\right]\;\;\;\;\;\;=\left(b-axz-cz-axz^{2}+z+\tau_{2}^{2}z\right)dt-\tau_{2}zdW_{2}+\tau_{3}zdW_{3}.$$

Then (2.2) is changed to

(2.3) $$dx=[rx(1-x-y)-axyz]dt+\tau_{1}x(1-x-y)dW_{1},\;dy=(axz-1)ydt+\tau_{2}ydW_{2},\;dz=\left[b+\left(1+\tau_{2}^{2}-ax-c\right)z-axz^{2}\right]dt-\tau_{2}zdW_{2}+\tau_{3}zdW_{3}.$$

We still denote by *u*(*t*) = (*x*(*t*), *y*(*t*), *z*(*t*)) the solution process of the system (2.3). The drift term and the diffusion term of (2.3) are also denoted by

$$f(u)=\left[\begin{array}{c} rx(1-x-y)-axyz\\ (axz-1)y\\ b+\left(1+\tau_{2}^{2}-ax-c\right)z-axz^{2} \end{array}\right]\text{,}\;\text{and}\;g(u)=\left[\begin{array}{ccc} \tau_{1}x(1-x-y) & 0 & 0\\ 0 & \tau_{2}y & 0\\ 0 & -\tau_{2}z & \tau_{3}z \end{array}\right].$$

The following theorem, that will be proved in Section 3, guarantees the global non-negativity of the solution of the system (2.3) for any positive initial value.

### Theorem 2.1.

*For any initial value $\left(x(0),y(0),z(0))\in {\mathbb{R}}_{+}^{3}:=\{(x,y,z):x\geq 0,y\geq 0,z\geq 0\right\}$, there exists a unique a.s. continuous global solution* (*x*(*t*), *y*(*t*), *z*(*t*))*, t* ≥ 0*, that remains in ${\mathbb{R}}_{+}^{3}$ a.s. In particular, if x*(0) = 0 *then x*(*t*) = 0 *for all t* > 0 *a.s. and if x*(0) > 0 *then x*(*t*) > 0 *for all t* > 0 *a.s. Similarly, if y*(0) = 0 *then y*(*t*) = 0 *for all t* > 0 *a.s. and if y*(0) > 0 *then y*(*t*) > 0 *for all t* > 0 *a.s. Finally, if z*(0) ≥ 0 *then z*(*t*) > 0 *for all t* > 0 *a.s. Furthermore, the solution* (*x*(*t*), *y*(*t*), *z*(*t*)) *is a strong Markov process that possesses the Feller property.*

Our analysis in Section 3 shows that there are only two ergodic invariant measures

$$\mu_{1}=\delta_{0}^{*}\times \delta_{0}^{*}\times \pi_{1}\;\text{and}\;\mu_{2}=\delta_{1}^{*}\times \delta_{0}^{*}\times \pi_{2}$$

of (2.3) on the boundary $\partial {\mathbb{R}}_{+}^{3}$. Here $\delta_{0}^{*}$ and $\delta_{1}^{*}$ are Dirac measures with mass at 0 and 1, respectively. The invariant measure *π*_1_ has the inverse gamma distribution:$\pi_{1}\sim \mathrm{IG}\left(\frac{2\left(c-1-\tau_{2}^{2}\right)}{\tau_{2}^{2}+\tau_{3}^{2}}+1,\;\frac{2b}{\tau_{2}^{2}+\tau_{3}^{2}}\right)$. The invariant measure *π*_2_ has the generalized inverse Gaussian distribution: *π*_2_ ~ GIG(*θ*, *χ*, *ψ*), where $\theta =\frac{2\left(1+\tau_{2}^{2}-a-c\right)}{\tau_{2}^{2}+\tau_{3}^{2}}-1$, $\psi =\frac{4a}{\tau_{2}^{2}+\tau_{3}^{2}}$, and $\chi =\frac{4b}{\tau_{2}^{2}+\tau_{3}^{2}}$.

To classify solutions of the system (2.3), we define two combined parameters as follows.

$$\lambda :=\sqrt{ab}\;R_{\theta }(w)-1-\frac{\tau_{2}^{2}}{2},\;\zeta :=2c-2-\tau_{2}^{2}+\tau_{3}^{2}$$

where $w=\frac{4\sqrt{ab}}{\tau_{2}^{2}+\tau_{3}^{2}}$, $R_{\theta }(w)=\frac{K_{\theta +1}(w)}{K_{\theta }(w)}$, and *K*_*θ*_(·) is the modified Bessel function of the third kind with index *θ* which is given by

$$K_{\theta }(\phi )=\frac{1}{2}\int_{0}^{\infty }x^{\theta -1}\exp\left\{-\frac{1}{2}\phi \left(x+\frac{1}{x}\right)\right\}dx,\phi >0.$$

With these parameters and their thresholds, we give the complete picture of the stochastic dynamics of the system (2.3). Our main result is stated in the following theorem that will be proved in Section 3.

### Theorem 2.2.

*Assume that the initial values u* = (*x*, *y*, *z*) *are in ${\mathbb{R}}_{+}^{3,{}^{\circ}}:=\{(x,y,z):x>0,y>0,z>0\}$ such that x* + *y* ≤ 1*. The complete classification of solutions of the system* (2.3) *is as follows.*

#### Case 1.

*When ζ* < 0*, there is only one ergodic invariant measure μ*_2_ *for solutions of* (2.3) *on the boundary $\partial {\mathbb{R}}_{+}^{3}$.*

- *If λ* < 0 *then x*(*t*) *converges to* 1 *a.s., y*(*t*) *converges to* 0 *a.s., and z*(*t*) *converges a.s. to π*_2_ *weakly.*
- *If λ* > 0 *then the solution u*(*t*) *is strongly stochastically persistent in the sense that the solution converges to its unique invariant probability measure μ*_3_ *supported by ${\mathbb{R}}_{+}^{3,{}^{\circ}}$.*

#### Case 2.

*When ζ* ≥ 0*, there are two ergodic invariant measures μ*_1_ *and μ*_2_ *for solutions of* (2.3) *on the boundary $\partial {\mathbb{R}}_{+}^{3}$.*

- *If λ* < 0 *and $\tau_{1}<\sqrt{2r}$, then x*(*t*) *converges to* 1 *a.s., y*(*t*) *converges to* 0 *a.s., and z*(*t*) *converges a.s. to π*_2_ *weakly.*
- *If λ* < 0 *and $\tau_{1}<\sqrt{2r}$, then solutions starting near the interior of supp*(*μ*_2_) *will tend to stay close and concentrate on supp*(*μ*_1_).
- *If λ* > 0 *and $\tau_{1}<\sqrt{2r}$, then the solution u*(*t*) *is strongly stochastically persistent.*
- *If λ* > 0 *and $\tau_{1}<\sqrt{2r}$, then x*(*t*) *and y*(*t*) *both converge a.s. to* 0.

### Proposition 2.1.

*The deterministic part of the system* (2.3) *has three possible nonnegative equilibrium solutions, $E_{1}=\left(0,0,\frac{b}{c-1}\right)$, $E_{2}=\left(1,0,\frac{1}{2a}\left((1-a-c)+\sqrt{{\left(1-a-c\right)}^{2}+4ab}\right)\right)$, and $E_{3}=\left(\frac{1}{a\bar{z}},\frac{r(1-/a\bar{z})}{r+a\bar{z}},\bar{z}\right)$ where $\bar{z}=\frac{b-1}{c}$. The ergodic invariant measures μ*_1_ *and μ*_2_ *correspond to E*_1_ *and E*_2_*, respectively, in the sense that the means of the distributions of μ*_1_ *and μ*_2_ *approaches E*_1_ *and E*_2_*, respectively, when* (*τ*_1_, *τ*_2_, *τ*_3_) *approaches* (0, 0, 0).

From the transformation of variables, the information about the system (2.2) can be obtained. We write them as the interpretation of our main theorem. We will give some medical interpretation of these results and compare with our study in [12].

### Interpretation 2.1.

*Consider the non-dimensionalized uninfected tumor cell population x*(*t*)*, infected tumor cell population y*(*t*)*, and free virus population v*(*t*) *start in ${\mathbb{R}}_{+}^{3,{}^{\circ}}:=\{(x,y,z):x>0,y>0,v>0\}$ such that x* + *y* ≤ 1*, which corresponds to the system* (2.2)*. Then, according to the thresholds ζ and λ, we can describe how each population will evolve as follows.*

#### Case 1.

*When ζ* < 0*, the tumor cannot be eradicated completely a.s.*

- *If λ* < 0 *then* (*x*(*t*), *y*(*t*), *v*(*t*)) converges to ${\bar{\mu }}_{2}=\delta_{1}^{*}\times \delta_{0}^{*}\times \delta_{0}^{*}$ a.s.
- *If λ* > 0 *then* (*x*(*t*), *y*(*t*), *v*(*t*)) *is strongly stochastically coexistence in the sense that* (*x*(*t*), *y*(*t*), *v*(*t*)) *converges to a unique invariant probability measure ${\bar{\mu }}_{3}$ supported by ${\mathbb{R}}_{+}^{3,{}^{\circ}}$.*

#### Case 2.

*When ζ* ≥ 0*, there is some possibilities to eradicate the tumor by oncolytic viruses.*

- *If λ* < 0 *and $\tau_{1}<\sqrt{2r}$, then* (*x*(*t*), *y*(*t*), *v*(*t*)) converges to ${\bar{\mu }}_{2}=\delta_{1}^{*}\times \delta_{0}^{*}\times \delta_{0}^{*}$ a.s.
- *If λ* > 0 *and $\tau_{1}<\sqrt{2r}$, then the solution* (*x*(*t*), *y*(*t*), *v*(*t*)) *is strongly stochastically coexistence.*
- *If λ* > 0 *and $\tau_{1}<\sqrt{2r}$, then* (*x*(*t*), *y*(*t*), *v*(*t*)) converge to ${\bar{\mu }}_{1}=\delta_{0}^{*}\times \delta_{0}^{*}\times \delta_{0}^{*}$ a.s.

Using the transformation of variables or directly deduce, we have a similar proposition as 2.1.

### Proposition 2.2.

*The deterministic part of the system* (2.2) *has three equilibrium solutions, Q*_1_ = (0, 0, 0)*, Q*_2_ = (1, 0, 0)*, and $Q_{3}=\left(\frac{1}{a\bar{z}},\frac{r(1-/a\bar{z})}{r+a\bar{z}},\frac{r(1-/a\bar{z})}{a+r/\bar{z}}\right)$. The system* (2.2) has three ergodic invariant probability measures ${\bar{\mu }}_{1}=\delta_{0}^{*}\times \delta_{0}^{*}\times \delta_{0}^{*}$, ${\bar{\mu }}_{2}=\delta_{1}^{*}\times \delta_{0}^{*}\times \delta_{0}^{*}$, and ${\bar{\mu }}_{3}$, which correspond to Q_1_*, Q*_2_*, Q*_3_*, respectively.*

In our study [12], we obtained asymptotic properties of the system (1.1). There are three equilibrium solutions *Q*_1_, *Q*_2_, and *Q*_3_. *Q*_1_ is always unstable for any positive values of parameters. *Q*_2_ is globally asymptotically stable when the virus burst size *b* is smaller a threshold value $b_{s_{1}}$, while it is unstable if *b* is greater than $b_{s_{1}}$. There is a second threshold value of the viral burst size $b_{s_{2}}$, and under the second threshold value and other conditions, *Q*_3_ is locally asymptotically stable. The system (1.1) undergoes Hopf bifurcations with three families of periodic solutions when the virus burst size passes the second threshold value $b_{s_{2}}$. It is interesting that *Q*_3_ can be approximated by $\left(O\left(\frac{1}{b}\right),\;O\left(\frac{1}{b}\right),\;\frac{r}{a}\right)$ when the viral burst size *b* is very big. After incorporating environmental noise and stochastic effects into the system (1.1), there are three invariant probability measures in which solutions will approach them under various conditions. We have two combined parameters *ζ* and *λ* to describe asymptotical properties of solutions to the systems (2.2) or (2.3) as in Theorem 2.2. However, we would like to understand these results from the original system or how environmental noise and stochastic effects change the dynamical behaviors of the original system (1.1). Then, we need to understand how these two combined parameters connect to original parameters and their biological meanings. We have a proposition about the parameter *λ*.

### Proposition 2.3.

*The parameter $\lambda =\sqrt{ab}\;R_{\theta }(w)-1-\frac{\tau_{2}^{2}}{2}$ is an increasing function of the virus burst size b. Also consider λ is a function of noise intensities τ*_2_ *and τ*_3_*, and set $\bar{\lambda }:=\lim_{\left(\tau_{2},\tau_{3}\right)\to (0,0)}\lambda$. Then $\bar{\lambda }=0$ if and only if $b=b_{s_{1}}=1+\frac{c}{a}$, $\bar{\lambda }<0$ if and only if $b<b_{s_{1}}$, and $\bar{\lambda }>0$ if and only if $b>b_{s_{1}}$; or simply, $\bar{\lambda }$ also is a increasing function of b.*

The parameter *ζ* combines infected tumor cell lysis rate *δ*, virus degradation rate *γ*, and their stochastic variation *τ*_2_ and *τ*_3_, which describes possibilities if the tumor can be eradicated. More specifically, $\zeta =2c-2-\tau_{2}^{2}+\tau_{3}^{2}=2\frac{T_{\delta }}{T_{\gamma }}-2-\tau_{2}^{2}+\tau_{3}^{2}$, where *T*_*δ*_ is the average life time of infected tumor cells, and *T*_*γ*_ is the average life time of free viruses in tumor tissue. *ζ* < 0 means $\frac{T_{\delta }}{T_{\gamma }}+\frac{\tau_{3}^{2}}{2}<\frac{\tau_{2}^{2}}{2}+1$. We may interpret that, if the ratio between the life time of infected tumor cells to the life time of free viruses is small and stochastic effects of viruses also is small comparing with stochastic effects of infected tumor cells, it is impossible to eradicate the tumor for viral therapy. However, in this situation, the viral therapy may partly success which depends on *λ*, or implicitly the outcome of the virotherapy depends on the virus burst size *b*. As in the deterministic model (1.1), if *b* is smaller than the threshold $b_{s_{1}}$ which corresponds to *λ* < 0 (it is deduced from continuity of *λ* as a function of *b*, *τ*_2_, and *τ*_3_), then the infected tumor cell population and virus population will disappear, and only tumor cell population is left a.s., or the system approaches the invariant probability measure ${\bar{\mu }}_{2}=\delta_{1}^{*}\times \delta_{0}^{*}\times \delta_{0}^{*}$. If *b* is greater than the threshold $b_{s_{1}}$ which corresponds to *λ* > 0, three populations will coexist, or the system approaches the invariant probability measure ${\bar{\mu }}_{3}$, in which we may say viral therapy achieve some partial success.

When *ζ* > 0 which means $\frac{T_{\delta }}{T_{\gamma }}+\frac{\tau_{3}^{2}}{2}>\frac{\tau_{2}^{2}}{2}+1$. We may interpret that, if the ratio between the life time of infected tumor cells to the life time of free viruses is big and stochastic effects of viruses also is big comparing with stochastic effects of infected tumor cells, there is some possibilities to eradicate the tumor by viral therapy. In this case, there is a third threshold value for stochastic variations of tumor cell growth *τ*_1_ that comes to play some roles. This value is $2r=2\frac{\rho }{\delta }$, scaled tumor cell growth rate. When *b* is smaller than the threshold $b_{s_{1}}$ which corresponds to *λ* < 0, the noise intensity *τ*_1_ or tumor cell variance $\tau_{1}^{2}$ is smaller than the double of the scaled tumor cell growth rate, then the viral treatment will complete fail. When *b* is greater than the threshold $b_{s_{1}}$ which corresponds to *λ* > 0, and the noise intensity $\frac{1}{2}\tau_{1}^{2}$ is not strong or smaller than scaled tumor cell growth rate, the system eventually will have three populations coexist, where the viral therapy reaches partial success. However, unlike in the corresponding deterministic model (1.1), when *b* is greater than the threshold $b_{s_{1}}$ which corresponds to *λ* > 0, and the noise intensity $\frac{1}{2}\tau_{1}^{2}$ is strong or greater than scaled tumor cell growth rate, the viral therapy will eradicate the tumor. A medical implication could be that viral therapy can success without too big virus burst size.

## 3. Analysis of the model

This section is devoted to proving results in Section 2.

### 3.1. Proof of Theorem 2.1

Consider the system (2.3). Since the drift term *f*(*u*) and the diffusion term *g*(*u*) are locally Lipschitz continuous, there exists a unique local a.s. continuous solution *u*(*t*) up to the explosion time

$$\tau_{e}=\inf\{t>0:\min\{x(t),y(t),z(t)\}=-\infty \;\text{or}\;\max\{x(t),y(t),z(t)\}=\infty \}.$$

Also, the solution *u*(*t*) = (*x*(*t*), *y*(*t*), *z*(*t*)), *t* ∈ (0, *τ*_*e*_), is a strong Markov process (see [24]). Denote by (*x*, *y*, *z*) the initial value of *u*(*t*). First, we will show that if (*x*, *y*, *z*) is in ${\mathbb{R}}_{+}^{3}$ then *u*(*t*) is also in ${\mathbb{R}}_{+}^{3}$ for all *t* ∈ (0, *τ*_*e*_) a.s.

From the equation of *x*(*t*), we get

$$x(t)=x\exp\{\int_{0}^{t}\left[r(1-x(s)-y(s))-ay(s)z(s)-\frac{\tau_{1}^{2}}{2}{\left(1-x(s)-y(s)\right)}^{2}\right]ds\;\;\;\;\;\;\;\;\;\;\;\;\;\;\;\;\;\;\;\;\;\;\;\;\;\;\;\;\;\;\;+\tau_{1}\int_{0}^{t}(1-x(s)-y(s))dW_{1}(s)\}.$$

So, if *x* = 0, then ${\mathbb{P}}_{0,y,z}\left\{x(t)=0\forall t\in \left(0,\tau_{e}\right)\right\}=1$ for all *y* ≥ 0 and *z* ≥ 0; if *x* > 0, then ${\mathbb{P}}_{x,y,z}\{x(t)>0\forall t\in \left(0,\tau_{e}\right)\}=1$ for all *y* ≥ 0 and *z* ≥ 0.

The second equation of (2.3) implies

$$y(t)=y\exp\left\{\int_{0}^{t}\left(ax(s)z(s)-1-\frac{\tau_{2}^{2}}{2}\right)ds+\tau_{2}W_{2}(t)\right\}.$$

If *y* = 0, then ${\mathbb{P}}_{x,0,z}\left\{y(t)=0\forall t\in \left(0,\tau_{e}\right)\right\}=1$ for all *x* ≥ 0 and *z* ≥ 0; if *y* > 0, then ${\mathbb{P}}_{x,y,z}\{y(t)>0\forall t\in \left(0,\tau_{e}\right)\}=1$ for all *x* ≥ 0 and *z* ≥ 0.

The last equation of (2.3) follows

$$z(t)=\phi (t)\left[z+\int_{0}^{t}b\phi^{-1}(s)ds\right]$$

where

$$\phi (t)=\exp\left\{\int_{0}^{t}\left[\left(1+\tau_{2}^{2}-ax(s)-c\right)-ax(s)z(s)-\frac{\tau_{2}^{2}}{2}-\frac{\tau_{3}^{2}}{2}\right]ds-\tau_{2}W_{2}(t)+\tau_{3}W_{3}(t)\right\}.$$

This implies that if *z* ≥ 0, then ${\mathbb{P}}_{x,y,z}\left\{z(t)>0\forall t\in \left(0,\tau_{e}\right)\right\}=1$ for all *x* ≥ 0 and *y* ≥ 0.

Hence, we have shown that if *x* ≥ 0, *y* ≥ 0, and *z* ≥ 0 then *x*(*t*) ≥ 0, *y*(*t*) ≥ 0, and *z*(*t*) ≥ 0 for all *t* ∈ (0, *τ*_*e*_) a.s.

Next, we show that *τ*_*e*_ = ∞ a.s. Consider *V*(*x*, *y*, *z*) = *x* + *y* + ln(1 + *z*). By Ito’s formula, for all *t* ∈ (0, *τ*_*e*_) we get

$$LV(t)=rx(t)[1-x(t)-y(t)-ax(t)y(t)z(t)+ax(t)y(t)z(t)-y(t)\;\;\;\;\;\;\;\;\;\;\;\;\;\;\;\;+\frac{b+\left(1+\tau_{2}^{2}-ax(t)-c\right)z(t)-ax(t)z^{2}(t)}{1+z(t)}-\frac{1}{2}\left(\tau_{2}^{2}+\tau_{3}^{2}\right)\frac{z^{2}(t)}{{\left(1+z(t)\right)}^{2}}\;\;\;\;\;\;\;\;\;\;\;\;\;\;\;\;\leq \left(b+1+\tau_{2}^{2}\right)1_{\left\{x+y>1\right\}}+\left(r+b+1+\tau_{2}^{2}\right)1_{\left\{x+y\leq 1\right\}}=:H.$$

Let *τ*_*n*_ := inf{*t* ∈ [0, *τ*_*e*_) : *x*(*t*) > *n* or *y*(*t*) > *n* or *z*(*t*) > *n*}. Clearly, *τ*_*n*_ increases to *τ*_∞_ as *n* → ∞ where

$$\tau_{\infty }:=\inf\left\{t\in \left[0,\tau_{e}\right):x(t)=\infty \;\text{or}\;y(t)=\infty \;\text{or}\;z(t)=\infty \right\}.$$

Since *τ*_∞_ ≤ *τ*_*e*_ a.s., it suffices to prove that ${\mathbb{P}}_{x,y,z}\left\{\tau_{\infty }=\infty \right\}=1$. Fix *t* > 0, Ito’s formula for *V* implies

$$E_{x,y,z}V\left(t\wedge \tau_{n}\right):=E_{x,y,z}V\left(x\left(t\wedge \tau_{n}\right),y\left(t\wedge \tau_{n}\right),z\left(t\wedge \tau_{n}\right)\right)\;\;\;\;\;\;\;\;\;\;\;\;\;\;\;\;\;\;\;\;\;\;\;\;\;\;\;\;\;\;\;\;\;\;\;\;\;=V(x,y,z)+E_{x,y,z}\int_{0}^{t\wedge \tau_{n}}LV(x(s),y(s),z(s))ds\;\;\;\;\;\;\;\;\;\;\;\;\;\;\;\;\;\;\;\;\;\;\;\;\;\;\;\;\;\;\;\;\;\;\;\;\;\leq K+H\left(t\wedge \tau_{n}\right)\leq K+Ht$$

where *K* := *V*(*x*, *y*, *z*). On the other hand,

$$E_{x,y,z}V\left(t\wedge \tau_{n}\right)\geq \int_{\left\{\tau_{n}<t\right\}}V\left(x\left(\tau_{n}\right),y\left(\tau_{n}\right),z\left(\tau_{n}\right)\right)d{\mathbb{P}}_{x,y,z}\geq (n\wedge \ln(1+n)){\mathbb{P}}_{x,y,z}\left\{\tau_{n}<t\right\}.$$

Thus

$${\mathbb{P}}_{x,y,z}\left\{\tau_{n}<t\right\}\leq \frac{K+Ht}{n\wedge \ln(1+n)}\to 0,\text{as}\;n\to \infty .$$

Since *t* > 0 is arbitrary, ${\mathbb{P}}_{x,y,z}\left\{\tau_{\infty }<\infty \right\}=0$ and hence *τ*_∞_ = ∞ a.s.

This completes the proof. □

### 3.2. Proof of Theorem 2.2

Before giving the detailed proof of the main theorem 2.2, we analyze solutions of the system (2.3) on the boundary $\partial {\mathbb{R}}_{+}^{3}$ firstly. When *x*(0) = 0, *x*(*t*) = 0 for all *t* ≥ 0 a.s. If *x* = 0 then the system (2.3) becomes

$$dY=-Ydt+\tau_{2}YdW_{2}$$

$$dZ=\left[b+\left(1+\tau_{2}^{2}-c\right)Z\right]dt-\tau_{2}ZdW_{2}+\tau_{3}ZdW_{3}.$$

The second equation for *y*(*t*) implies

$$Y(t)=Y(0)\exp\left\{-\left(1+\frac{\tau_{2}^{2}}{2}\right)t+\tau_{2}W_{2}(t)\right\}.$$

So *Y*(*t*) → 0 a.s. for all *Y*(0) = *y*(0) ≥ 0. Consider the last equation for *Z*

(3.1) $$dZ=\left[b-\left(c-1-\tau_{2}^{2}\right)Z\right]dt-\tau_{2}ZdW_{2}+\tau_{3}ZdW_{3}.$$

Fix *α*_1_ > 0, consider

$$s(Z)=\int_{\alpha_{1}}^{Z}\exp\left\{-\int_{\alpha_{1}}^{y}\frac{2b-2\left(c-1-\tau_{2}^{2}\right)u}{\left(\tau_{2}^{2}+\tau_{3}^{2}\right)u^{2}}du\right\}dy\;\;\;\;\;\;\;\;=C_{1}\int_{\alpha_{1}}^{Z}y^{2\left(c-1-\tau_{2}^{2}\right)/\left(\tau_{2}^{2}+\tau_{3}^{2}\right)}\exp\left\{\frac{2b}{\left(\tau_{2}^{2}+\tau_{3}^{2}\right)y}\right\}dy$$

where *C*_1_ is some positive constant. Rewrite the integrand as

$$y^{-2\left(1+\tau_{2}^{2}-c\right)/\left(\tau_{2}^{2}+\tau_{3}^{2}\right)}\left[1+\frac{2b}{\tau_{2}^{2}+\tau_{3}^{2}}\frac{1}{y}+\frac{1}{2!}\frac{4b^{2}}{{\left(\tau_{2}^{2}+\tau_{3}^{2}\right)}^{2}}\frac{1}{y^{2}}+\cdots \right]$$

Since there exists a $k\in Z_{+}$ such that $-\frac{2\left(1+\tau_{2}^{2}-c\right)}{\tau_{2}^{2}+\tau_{3}^{2}}-k<-1,\;s(0+)=-\infty$. If $\zeta =2c-2-\tau_{2}^{2}+\tau_{3}^{2}<0$, then $-\frac{2\left(1+\tau_{2}^{2}-c\right)}{\tau_{2}^{2}+\tau_{3}^{2}}+1<0$, and, so *s*(∞) < ∞. By the item 2 of Theorem 3.1 on page 447 in [25], $\underset{t\to \infty }{\lim}Z(t)=\infty$ a.s. In this case, (3.1) does not have any invariant measure. If *ζ* ≥ 0, then $-\frac{2\left(1+\tau_{2}^{2}-c\right)}{\tau_{2}^{2}}+1\geq 0$, and this implies that *s*(∞) = ∞. Then *Z*(*t*) oscillates between 0 and ∞. Then (3.1) has a unique invariant measure $\pi_{1}\sim \text{IG}\left(\frac{2\left(c-1-\tau_{2}^{2}\right)}{\tau_{2}^{2}+\tau_{3}^{2}}+1,\frac{2b}{\tau_{2}^{2}+\tau_{3}^{2}}\right)$ (the inverse gamma distribution with parameters $\frac{2\left(c-1-\tau_{2}^{2}\right)}{\tau_{2}^{2}+\tau_{3}^{2}}+1$ and $\frac{2b}{\tau_{2}^{2}+\tau_{3}^{2}}$).

When *x*(0) > 0, *x*(*t*) > 0 for all *t* > 0 a.s. If *y*(0) = 0, then the second equation of (2.3) implies *y*(*t*) = 0 for all *t* > 0 a.s. So, when *y* = 0, the equation for *x* becomes

(3.2) $$d\tilde{x}=r\tilde{x}(1-\tilde{x})dt+\tau_{1}\tilde{x}(1-\tilde{x})dW_{1}.$$

Fix *α*_2_ > 0, we compute

$$s(\tilde{x})=\int_{\alpha_{2}}^{\tilde{x}}\exp\left\{-\int_{\alpha_{2}}^{y}\frac{2ru(1-u)}{\tau_{1}^{2}u^{2}{\left(1-u\right)}^{2}}du\right\}dy\;\;\;\;\;\;\;\;\;\;\;=C_{2}\int_{\alpha_{2}}^{\tilde{x}}{\left(\frac{1}{y}-1\right)}^{2r/\tau_{1}^{2}}dy$$

where *C*_2_ is some positive constant. Clearly, *s*(1−) < ∞. Since $\lim_{y\to 0+}{\left(\frac{1}{y}-1\right)}^{2r/\tau_{1}^{2}}=\infty$, for any *M* > 0, there exists a 0 < *δ* < *α*_2_ so that, if 0 < *y* < *δ*, then ${\left(\frac{1}{y}-1\right)}^{2r/\tau_{1}^{2}}\geq \frac{M}{\delta C_{2}}$. But we have

$$s(0+)=-C_{2}\int_{0}^{\alpha_{2}}{\left(\frac{1}{y}-1\right)}^{2r/\tau_{1}^{2}}dy\;\;\;\;\;\;\;\;\;\;\;=-C_{2}\int_{0}^{\delta }{\left(\frac{1}{y}-1\right)}^{2r/\tau_{1}^{2}}dy-C_{2}\int_{\delta }^{\alpha_{2}}{\left(\frac{1}{y}-1\right)}^{2r/\tau_{1}^{2}}dy\;\;\;\;\;\;\;\;\;\;\;\leq -C_{2}\int_{0}^{\delta }{\left(\frac{1}{y}-1\right)}^{2r/\tau_{1}^{2}}dy\leq -C_{2}\delta \frac{M}{\delta C_{2}}=-M.$$

Letting *M* → ∞, it gives *s*(0+) = −∞. This means that $\lim_{t\to \infty }\tilde{x}(t)=1$ a.s. for any $\tilde{x}(0)=x(0)>0$. When *x* = 1 and *y* = 0, the last equation for *z* becomes

(3.3) $$d\tilde{z}=\left[b+\left(1+\tau_{2}^{2}-a-c\right)\tilde{z}-a{\tilde{z}}^{2}\right]dt-\tau_{2}\tilde{z}dW_{2}+\tau_{3}\tilde{z}dW_{3}.$$

Fix *α* > 0, consider

$$s(\tilde{z})=\int_{\alpha }^{\tilde{z}}\exp\left\{-\int_{\alpha }^{y}\frac{2b+2\left(1+\tau_{2}^{2}-a-c\right)u-2au^{2}}{\left(\tau_{2}^{2}+\tau_{3}^{2}\right)u^{2}}du\right\}dy\;\;\;\;\;\;\;\;\;\;=C_{3}\int_{\alpha }^{\tilde{z}}y^{-2\left(1+\tau_{2}^{2}-a-c\right)/\left(\tau_{2}^{2}+\tau_{3}^{2}\right)}\exp\left\{\frac{2b}{\tau_{2}^{2}+\tau_{3}^{2}}y^{-1}+\frac{2a}{\tau_{2}^{2}+\tau_{3}^{2}}y\right\}dy$$

where *C*_3_ is some positive constant. The integrand can be written as

$$y^{-2\left(1+\tau_{2}^{2}-a-c\right)/\left(\tau_{2}^{2}+\tau_{3}^{2}\right)}[1+\left(\frac{2b}{\tau_{2}^{2}+\tau_{3}^{2}}\frac{1}{y}+\frac{2a}{\tau_{2}^{2}+\tau_{3}^{2}}y\right)\;\;\;\;\;\;\;\;\;\;\;\;\;\;\;\;\;\;\;\;\;\;\;\;\;\;\;\;\;\;\;\;\;\;\;\;\;\;\;\;\;+\left(\frac{4b^{2}}{{\left(\tau_{2}^{2}+\tau_{3}^{2}\right)}^{2}}\frac{1}{y^{2}}+\frac{8ab}{{\left(\tau_{2}^{2}+\tau_{3}^{2}\right)}^{2}}+\frac{4a^{2}}{{\left(\tau_{2}^{2}+\tau_{3}^{2}\right)}^{2}}y^{2}\right)+\cdots ].$$

Clearly, there are *k*_1_ and *k*_2_ in $Z_{+}$ such that

$$-\frac{2\left(1+\tau_{2}^{2}-a-c\right)}{\tau_{2}^{2}+\tau_{3}^{2}}-k_{1}<-1\;\text{and}\;-\frac{2\left(1+\tau_{2}^{2}-a-c\right)}{\tau_{2}^{2}+\tau_{3}^{2}}+k_{2}>-1.$$

Hence *s*(0+) = −∞ and *s*(∞) = ∞. So $\tilde{z}(t)$ oscillates between 0 and ∞, and thus (3.3) has a unique invariant measure *π*_2_ ~ GIG(*θ*, *χ*, *ψ*), which is the generalized inverse Gaussian distribution with parameters θ∈ℝ, *χ* > 0, and *ψ* > 0 (see the Appendix), whose density takes the form

$$p(\tilde{z})=\frac{{\left(a/b\right)}^{\theta /2}}{2K_{\theta }\left(4\sqrt{ab}/\left(\tau_{2}^{2}+\tau_{3}^{2}\right)\right)}{\tilde{z}}^{\theta -1}\exp\left\{-\frac{1}{2}\left(\chi {\tilde{z}}^{-1}+\psi \tilde{z}\right)\right\},\tilde{z}\in (0,\infty ),$$

where $\theta :=\frac{2\left(1+\tau_{2}^{2}-a-c\right)}{\tau_{2}^{2}+\tau_{3}^{2}}-1\;$, $\chi :=\frac{4b}{\tau_{2}^{2}+\tau_{3}^{2}}$, and $\psi :=\frac{4a}{\tau_{2}^{2}+\tau_{3}^{2}};K_{\theta }(\cdot )$ is the modified Bessel function of the third kind with index *θ*. By law of large numbers,

$$\underset{t\to \infty }{\lim}\frac{1}{t}\int_{0}^{t}\tilde{z}(s)ds=\int_{0}^{\infty }\tilde{z}\pi_{2}(d\tilde{z})=R_{\theta }(w)\sqrt{b/a},$$

in which $R_{\theta }(w):=\frac{K_{\theta +1}(w)}{K_{\theta }(w)}$ and $w:=\frac{4\sqrt{ab}}{\tau_{2}^{2}+\tau_{3}^{2}}$.

In summary, on the boundary $\partial {\mathbb{R}}_{+}^{3}$

- If $\zeta =2c-2-\tau_{2}^{2}+\tau_{3}^{2}<0$, then the system (2.3) has only one invariant measure $\mu_{2}:=\delta_{1}^{*}\times \delta_{0}^{*}\times \pi_{2}$.
- If *ζ* ≥ 0 then the system (2.3) has two invariant measures $\mu_{1}:=\delta_{0}^{*}\times \delta_{0}^{*}\times \pi_{1}$ and *μ*_2_.

Note that

$$\int_{\partial {\mathbb{R}}_{+}^{3}}\left(axz-1-\frac{\tau_{2}^{2}}{2}\right)d\mu_{1}=-1-\frac{\tau_{2}^{2}}{2}<0,$$

$$\int_{\partial {\mathbb{R}}_{+}^{3}}\left(axz-1-\frac{\tau_{2}^{2}}{2}\right)d\mu_{2}=\sqrt{ab}R_{\theta }(w)-1-\frac{\tau_{2}^{2}}{2}.$$

We define a combined parameter as the threshold

$$\lambda :=\sqrt{ab}R_{\theta }(w)-1-\frac{\tau_{2}^{2}}{2},$$

and define the family of the random normalized occupation measures

$$\Pi_{t}(\cdot ):=\frac{1}{t}\int_{0}^{t}1_{\left\{u(s)\in \cdot \right\}}ds,t>0.$$

Then, we have the following claim.

#### Claim 3.1.

*Assume that λ* < 0*. For any initial value u* = (*x*, *y*, *z*) *in ${\mathbb{R}}_{+}^{3,{}^{\circ}}$ satisfying x* + *y* ≤ 1*, if Π*_*t*_(·) *converges weakly to μ*_2_ *a.s. and y*(*t*) *converges a.s. to* 0 *exponentially fast with the rate λ, then x*(*t*) *converges a.s. to* 1 *and z*(*t*) *converges weakly to π*_2_.

In order to prove Claim 3.1, we will utilize the non-negative semi-martingale convergence theorem (see Theorem 3.1 in [24]) and the following two lemmas, whose proofs will be given in the end of this subsection.

#### Lemma 3.1.

*If u*(*t*) = (*x*(*t*), *y*(*t*), *z*(*t*)) *is the solution of* (2.3) *with the initial value u* = (*x*, *y*, *z*) *satisfying x* > 0*, y* > 0*, z* > 0*, and x* + *y* ≤ 1 *then* 0 < *x*(*t*) < 1 *for all t* > 0 *a.s.*

#### Lemma 3.2.

Suppose the assumption of Lemma 3.1 is satisfied. Then $\underset{t\to \infty }{\lim\;\sup}E_{u}x(t)z(t)<\infty$.

##### Proof of Claim 3.1.

From the equation for *x*(*t*) of (2.3), we get

$$1-x(t)=1-x+\int_{0}^{t}x(s)y(s)[r+az(s)]ds-\int_{0}^{t}rx(s)[1-x(s)]ds\;\;\;\;\;\;\;\;\;\;\;\;\;\;\;\;\;\;-\int_{0}^{t}\tau_{1}x(s)[1-x(s)-y(s)]dW_{1}(s).$$

Denote

$$A_{t}:=\int_{0}^{t}x(s)y(s)[r+az(s)]ds,U_{t}:=\int_{0}^{t}rx(s)[1-x(s)]ds,$$

$$M_{t}:=-\int_{0}^{t}\tau_{1}x(s)[1-x(s)-y(s)]dW_{1}(s).$$

Clearly, *A*_*t*_ and *U*_*t*_ are continuous adapted ($F_{t}$-measurable) increasing processes with *A*_0_ = *U*_0_ = 0. *M*_*t*_ is a local martingale with *M*_0_ = 0 and 1 − *x*(*t*) ≥ 0 a.s. by Lemma 3.1.

Then, we show that lim_*t*→∞_ *A*_*t*_ < ∞ a.s. Since $\underset{t\to \infty }{\lim}\frac{\ln y(t)}{t}=\lambda <0$, there is a Θ > 0 such that *t* ≥ Θ implies $y(t)\leq \exp\left\{\frac{\lambda t}{2}\right\}$. But, then for *t* ≥ Θ

$$\int_{0}^{t}rx(s)y(s)ds=\int_{0}^{\Theta }rx(s)y(s)ds+\int_{\Theta }^{t}rx(s)y(s)ds\;\;\;\;\;\;\;\;\;\;\;\;\;\;\;\;\;\;\;\;\;\;\;\;\;\;\;\;\;\;\;\;\leq r\Theta +r\int_{\Theta }^{t}\exp\left\{\frac{\lambda s}{2}\right\}ds=r\Theta +\frac{2r}{-\lambda }\left[\exp\left\{\frac{\lambda \Theta }{2}\right\}-\exp\left\{\frac{\lambda t}{2}\right\}\right],$$

which follows that $\underset{t\to \infty }{\lim}\int_{0}^{t}rx(s)y(s)ds<\infty$ a.s. On the other hand, by Lemma 3.2, we can use the Markov inequality to show $N:=\underset{t\geq 0}{\sup\;}x(t)z(t)<\infty$ a.s. So the same argument as above implies $\underset{t\to \infty }{\lim}\int_{0}^{t}ax(s)y(s)z(s)ds<\infty$ a.s. Therefore $\underset{t\to \infty }{\lim}A_{t}<\infty$ a.s.

By Theorem 3.9 on page 14 in [24], $\underset{t\to \infty }{\lim}(1-x(t))<\infty$ a.s. and $\underset{t\to \infty }{\lim}\int_{0}^{t}x(s)[1-x(s)]ds<\infty$ a.s.

If *x*(*t*)[1 − *x*(*t*)] did not converge a.s. to 0, then there would be an Ω_1_ ⊆ Ω with $\mathbb{P}\left(\Omega_{1}\right)>0$ so that $\underset{t\to \infty }{\lim\;\inf\;}x(t,\omega )[1-x(t,\omega )]=p(\omega )>0$ for all ω ∈ Ω_1_. Fix ω ∈ Ω_1_, there exists a *T* := *T*(ω) > 0 so that *t* ≥ *T* implies $x(t,\omega )[1-x(t,\omega )]>\frac{1}{2}p(\omega )$. Hence

$$\int_{0}^{\infty }x(s,\omega )[1-x(s,\omega )]ds\geq \int_{T}^{\infty }x(s,\omega )[1-s(s,\omega )]ds\;\;\;\;\;\;\;\;\;\;\;\;\;\;\;\;\;\;\;\;\;\;\;\;\;\;\;\;\;\;\;\;\;\;\;\;\;\;\;\;\;\;\;\;\;\;\;\;\;\;\;\;\;\;\geq \frac{1}{2}p(\omega )\int_{T}^{\infty }ds=\infty .$$

Then Ω_1_ ⊆ Ω_2_, where $\Omega_{2}=\left\{\omega ;\int_{0}^{\infty }x(s,\omega )[1-x(s,\omega )]ds=\infty \right\}$. This implies that $\mathbb{P}\left(\Omega_{2}\right)>0$. But this contradicts the fact that $\underset{t\to \infty }{\lim}\int_{0}^{t}x(s)[1-x(s)]ds<\infty$ a.s. Therefore

(3.4) $$\underset{t\to \infty }{\lim}x(t)[1-x(t)]=0\;a.s.$$

Since *Π*_*t*_(·) converges weakly to *μ*_2_, there exists a sequence {*t*_*k*_}_*k*≥1_ such that *t*_*k*_ ↑ ∞ and

$$\underset{k\to \infty }{\lim}\int_{D}xP\left(t_{k},u,du\right)=\int_{D}x\mu_{2}(du)=1,$$

where *P*(*t*, *u*, ·) is the transition probability of the solution *u*(*t*) of the system (2.3). In other words,$E_{u}x\left(t_{k}\right)\to 1$. Combining this fact with (3.4), we can conclude that *x*(*t*) converges a.s. to 1. Moreover, since

$$\underset{k\to \infty }{\lim}\int_{D}|z-\tilde{z}|P\left(t_{k},u,du\right)=\int_{D}|z-\tilde{z}|\mu_{2}(du)=0,$$

$E_{u}\left|z\left(t_{k}\right)-\tilde{z}\left(t_{k}\right)\right|\to 0$ As $\tilde{z}(t)$

Now, we give a proof of our main theorem 2.2. Notice that Assumptions 1.1–1.5 and Theorems 1.1 and 1.3 mentioned in the proof are referred in [23], since our proof is based on Theorem 1.1 and Theorem 1.3 there.

##### Proof of Theorem 2.2.

First, we denote *x*_1_ = *x*, *x*_2_ = *y*, *x*_3_ = *z*, *f*_1_ = *r*(1 − *x* − *y*) − *ayz*, *f*_2_ = *axz* − 1, $f_{3}=\frac{b}{z}+1+\tau_{2}^{2}-ax-c-axz$, *g*_1_ = 1 − *x* − *y*, *g*_2_ = *g*_3_ = 1, and

$$\Gamma ={\left(\sigma_{ij}\right)}_{1\leq i,j\leq 3}=\left[\begin{array}{ccc} \tau_{1} & 0 & 0\\ 0 & \tau_{2} & 0\\ 0 & -\tau_{2} & \tau_{3} \end{array}\right].$$

It is clear that *f*_*i*_ and *g*_*i*_ (*i* = 1, 2, 3) are locally Lipschitz. For *c* = (0, 0, 1)^*T*^, *u* = (*x*, *y*, *z*)^*T*^, and *γ*_*b*_ > 0, we have

$$\;\;\;\;\;\;\;\;\;\;\;\;\;\;\;\;\;\;\;\;\frac{\sum_{i=1}^{3}c_{i}x_{i}f_{i}}{1+c^{T}u}=\frac{b+\left(1+\tau_{2}^{2}-ax-c\right)z-axz^{2}}{1+z},\;\frac{-\frac{1}{2}\sum_{i,j=1}^{3}\sigma_{ij}c_{i}c_{j}x_{i}x_{j}g_{i}g_{j}}{{\left(1+c^{T}u\right)}^{2}}=-\frac{1}{2}\frac{\tau_{2}^{2}z^{2}+\tau_{3}^{2}y^{2}z^{2}}{{\left(1+z\right)}^{2}},\text{and}\;\gamma_{b}\left[1+\sum_{i=1}^{3}\left|f_{i}\right|+\sum_{i=1}^{3}g_{i}^{2}\right]=\gamma_{b}[1+|r(1-x-y)-ayz|+|axz-1|\;\;\;\;\;\;\;\;\;\;\;\;\;\;\;\;\;\;\;\;\;\;\;\;\;\;\;\;\;\;\;\;\;\;\;\;\;\;\;\;\;\;\;\;\;\;\;\;\;\;\;\;+\left|b/z+1+\tau_{2}^{2}-ax-c-axz\right|+2+{\left(1-x-y\right)}^{2}].$$

Note that, since 0 ≤ *x*(*t*) ≤ 1 for all *t* ≥ 0 a.s. by Lemma 3.1, we can show that $E_{u}(x(t)+y(t))\leq 1$. This implies that $E_{u}y(t)\leq 1$ and hence, by Markov’s inequality, we can prove that *y*(*t*) is bounded a.s. Thus

$$\frac{\sum_{i=1}^{3}c_{i}x_{i}f_{i}}{1+c^{T}u}-\frac{\frac{1}{2}\sum_{i,j=1}^{3}\sigma_{ij}c_{i}c_{j}x_{i}x_{j}g_{i}g_{j}}{{\left(1+c^{T}u\right)}^{2}}+\gamma_{b}\left[1+\sum_{i=1}^{3}\left|f_{i}\right|+\sum_{i=1}^{3}g_{i}^{2}\right]\;\leq \frac{b}{1+z}+\frac{\left(1+\tau_{2}^{2}-ax-c\right)z}{1+z}-\frac{axz^{2}}{1+z}+\frac{\gamma_{b}b}{z}+\gamma_{b}K_{4}(1+z)$$

for some constant *K*_4_ > 0. When *z* is large enough, we can choose *γ*_*b*_ > 0 sufficiently small so that

$$\frac{b}{1+z}+\frac{\left(1+\tau_{2}^{2}-ax-c\right)z}{1+z}-\frac{axz^{2}}{1+z}+\frac{\gamma_{b}b}{z}+\gamma_{b}K_{4}(1+z)<0.$$

This shows that

$$\underset{\|u\|\to \infty }{\lim}\left\{\frac{\sum_{i=1}^{3}c_{i}x_{i}f_{i}}{1+c^{T}u}-\frac{\frac{1}{2}\sum_{i,j=1}^{3}\sigma_{ij}c_{i}c_{j}x_{i}x_{j}g_{i}g_{j}}{{\left(1+c^{T}u\right)}^{2}}+\gamma_{b}\left[1+\sum_{i=1}^{3}\left|f_{i}\right|+\sum_{i=1}^{3}g_{i}^{2}\right]\right\}<0$$

where ‖*u*‖ := |*x*| + |*y*| + |*z*|. Moreover, it is easy to compute

$$\mathrm{diag}\left(g_{1},g_{2},g_{3}\right)\Gamma \Gamma^{T}\mathrm{diag}\left(g_{1},g_{2},g_{3}\right)=\left[\begin{array}{ccc} \tau_{1}^{2}{\left(1-x-y\right)}^{2} & 0 & 0\\ 0 & \tau_{2}^{2} & \tau_{2}^{2}\\ 0 & \tau_{2}^{2} & \tau_{3}^{2}+\tau_{2}^{2} \end{array}\right]$$

which is positive definite for all $(x,y,z)\in {\mathbb{R}}_{+}^{3,{}^{\circ}}$ satisfying *x* + *y* ≤ 1. Thus, Assumption 1.1 in [23] is fulfilled for the system (2.3).

Next, let M be the set of ergodic invariant measures of the system (2.3) supported by the boundary $\partial {\mathbb{R}}_{+}^{3}$. Consider two cases.

###### Case 1.

$\zeta =2c-2-\tau_{2}^{2}+\tau_{3}^{2}<0$. There is only one ergodic invariant measure on $\partial {\mathbb{R}}_{+}^{3}$, which is $\mu_{2}=\delta_{1}^{*}\times \delta_{0}^{*}\times \pi_{2}$. Observe that

$$D_{\mu_{2}}:=\mathrm{supp}\left(\mu_{2}\right)=\left\{(x,y,z)\in {\mathbb{R}}_{+}^{3}:y=0\right\},$$

$$I_{\mu_{2}}=\{1,3\},\;I_{\mu_{2}}^{c}=\{2\},$$

$\lambda_{1}\left(\mu_{2}\right)=\lambda_{3}\left(\mu_{2}\right)=0,$ (by Lemma 2.1 in [23])

$$\lambda_{2}\left(\mu_{2}\right)=\sqrt{ab}R_{\theta }(w)-1-\frac{\tau_{2}^{2}}{2}=:\lambda .$$

Then $M=\left\{\mu_{2}\right\}$, and so $\mathrm{Conv}(M)=\left\{\mu_{2}\right\}$ (the convex hull of M, that is the set of probability measure *π* of the form $\pi (\cdot )=\sum_{\mu \in M}p_{\mu }\mu (\cdot )$ with $\sum_{\mu \in M}p_{\mu }=1,\;p_{\mu }\geq 0$. If *λ* > 0, then Assumption 1.2 holds and thus, by Theorem 1.1, the solution of (2.3) is strongly stochastically persistent. If *λ* < 0 then Assumption 1.3 holds. Note that

$$M_{\mu_{2}}:=\left\{v\in M:\mathrm{supp}(v)\subseteq \partial {\mathbb{R}}_{+}^{3}\right\}=\emptyset .$$

So

$$M^{1}:=\{\mu \in M:\mu \;\text{satisfies}\;\text{Assumption}\;1.3\}=\left\{\mu_{2}\right\}\neq \emptyset$$

and hence $M^{2}:=M\backslash M^{1}=\emptyset$. This means that Assumption 1.5 is satisfied. Furthermore, since $\sum_{i=1}^{3}g_{i}^{2}=2+{\left(1-x-y\right)}^{2}$ is bounded, for any 0 < *δ*_1_ < 1 we have

$$\underset{\|u\|\to \infty }{\lim}\frac{\left\|u{\|}^{\delta_{1}}\sum_{i=1}^{3}g_{i}^{2}(u\right)}{1+\sum_{i=1}^{3}\left(\left|f_{i}(u)\right|+{\left|g_{i}(u)\right|}^{2}\right)}=0.$$

Thus Assumption 1.4 is fulfilled. By Theorem 1.3, for any initial value (*x*, *y*, *z*) in ${\mathbb{R}}_{+}^{3,{}^{\circ}}$ satisfying *x* + *y* ≤ 1, *Π*_*t*_(·) converges weakly to the unique invariant measure *μ*_2_ and $\lim_{t\to \infty }\frac{\ln y(t)}{t}=\lambda$ w.p.1. By Claim 3.1, *x*(*t*) converges a.s. to 1 and *z*(*t*) converges weakly to *π*_2_ a.s.

###### Case 2.

*ζ* ≥ 0. There are two ergodic invariant measures on $\partial {\mathbb{R}}_{+}^{3}$, which are *μ*_1_ and *μ*_2_. It is straightforward to see that

$$D_{\mu_{1}}:=\mathrm{supp}\left(\mu_{1}\right)=\left\{(x,y,z)\in {\mathbb{R}}_{+}^{3}:x=0,y=0\right\}\subseteq D_{\mu_{2}},$$

$$I_{\mu_{1}}=\{3\},I_{\mu_{1}}^{c}=\{1,2\},$$

$$\lambda_{1}\left(\mu_{1}\right)=r-\frac{\tau_{1}^{2}}{2},\lambda_{2}\left(\mu_{1}\right)=-1-\frac{\tau_{2}^{2}}{2}$$

$\lambda_{3}\left(\mu_{1}\right)=0$ (by Lemma 2.1 in [23]).

Then $M=\left\{\mu_{1},\mu_{2}\right\}$ and so

$$\mathrm{Conv}(M)=\left\{\mu =p_{1}\mu_{1}+p_{2}\mu_{2}:p_{1}+p_{2}=1,p_{1}\geq 0,p_{2}\geq 0\right\}.$$

If $\tau_{1}<\sqrt{2r}$ and *λ* > 0 then *λ*_1_(*μ*_1_) > 0 and *λ*_2_(*μ*_2_) > 0. It is clear that, for any μ∈Conv(M), max{*λ*_1_(*μ*), *λ*_2_(*μ*), *λ*_3_(*μ*)} > 0. Then Assumption 1.2 holds and hence, by Theorem 1.1, the solution of (2.3) is strongly stochastically persistent.

If $\tau_{1}<\sqrt{2r}$ and *λ* < 0 then $M^{1}=\left\{\mu_{2}\right\}$. Since $M_{\mu_{2}}=\left\{\mu_{1}\right\}$ and $\mathrm{Conv}\left(M_{\mu_{2}}\right)=\left\{\mu_{1}\right\}$, max{*λ*_1_(*μ*_1_), *λ*_3_(*μ*_1_)} > 0. Hence Assumption 1.3 holds. Since $M^{2}=\left\{\mu_{1}\right\}$, $\mathrm{Conv}\left(M^{2}\right)=\left\{\mu_{1}\right\}$ and so $\underset{i=1,2,3}{\max}\;\lambda_{i}\left(\mu_{1}\right)>0$. Then Assumptions 1.4 and 1.5 are satisfied. By Theorem 1.3 and Claim 3.1, *x*(*t*) converges a.s. to 1, *y*(*t*) converges a.s. to 0, and *z*(*t*) converges weakly to *π*_2_ a.s.

If $\tau_{1}>\sqrt{2r}$ and *λ* > 0 then, since $\underset{i\in I_{\mu_{1}}^{c}}{\max}\;\lambda_{i}\left(\mu_{1}\right)<0$ and $M_{\mu_{1}}=\emptyset$, $M^{1}=\left\{\mu_{1}\right\}$ and so $M^{2}=\left\{\mu_{2}\right\}$. As $\underset{i=1.2.3}{\max}\;\lambda_{i}\left(\mu_{2}\right)=\lambda >0$, so Assumption 1.5 are satisfied. With the same argument as Case 1, we also have Assumption 1.4 is fulfilled. Thus, by Theorem 1.3, we can conclude that *x*(*t*) and *y*(*t*) both converge a.s. to 0 with the rates *λ*_1_(*μ*_1_) and *λ*_2_(*μ*_1_), respectively.

Lastly, if $\tau_{1}>\sqrt{2r}$ and *λ* < 0, then $\underset{i\in I_{\mu_{1}}^{c}}{\max}\;\lambda_{i}\left(\mu_{1}\right)<0$ and $\underset{i\in I_{\mu_{2}}^{c}}{\max}\;\lambda_{i}\left(\mu_{2}\right)<0$. However, the condition (1.7) in Assumption 1.3 does not hold because $\underset{i\in I_{\mu_{1}}}{\max}\;\lambda_{i}\left(\mu_{1}\right)<0$ and $\underset{i\in I_{\mu_{2}}}{\max}\lambda_{i}\left(\mu_{2}\right)=0$. This means that *μ*_1_ and *μ*_2_ are not repellers. In this case, solutions starting near the interior of $D_{\mu_{2}}$ will stay close and concentrate on $D_{\mu_{1}}$.

This completes the proof. □

##### Proof of Lemma 3.1.

Take *V*(*x*, *y*, *z*) = 2 − *x* − *y* − ln(1 − *x*) for *x* > 0, *y* > 0, *z* > 0 and *x* + *y* ≤ 1. By Ito’s formula, for all *x* > 0, *y* > 0, *z* > 0, and *x* + *y* ≤ 1 we have

$$LV=-rx(1-x-y)+axyz+\frac{rx(1-x-y)}{1-x}-\frac{axyz}{1-x}\;\;\;\;\;\;\;\;\;\;+\frac{\tau_{1}^{2}}{2}\frac{x^{2}{\left(1-x-y\right)}^{2}}{{\left(1-x\right)}^{2}}-axyz+y\leq rx+y+\frac{\tau_{1}^{2}x^{2}}{2}\leq K_{1}V(x,y,z)$$

for some suitable positive constant *K*_1_. Let *ζ*_*k*_ = inf{*t* ≥ 0 : *V*(*x*(*t*), *y*(*t*), *z*(*t*)) ≥ *k*} and fix *t* > 0. Then Ito’s formula implies

$$E_{x,y,z}V(t):=E_{x,y,z}V\left(x\left(\zeta_{k}\wedge t\right),y\left(\zeta_{k}\wedge t\right),z\left(\zeta_{k}\wedge t\right)\right)\;\;\;\;\;\;\;\;\;\;\;\;\;\;\;\;\;\;\;\;\;\;\;\;=V(x,y,z)+E_{x,y,z}\int_{0}^{\zeta_{k}\wedge t}LV(x(s),y(s),z(s))ds\;\;\;\;\;\;\;\;\;\;\;\;\;\;\;\;\;\;\;\;\;\;\;\;\leq V(x,y,z)+K_{1}\int_{0}^{t}E_{x,y,z}V\left(x\left(\zeta_{k}\wedge s\right),y\left(\zeta_{k}\wedge s\right),z\left(\zeta_{k}\wedge s\right)\right)ds.$$

By Gronwall’s inequality,$E_{x,y,z}V(t)\leq V(x,y,z)\exp\left\{K_{1}t\right\}$. But, since

$$E_{x,y,z}V(t)\geq \int_{\left\{\zeta_{k}\leq t\right\}}V\left(x\left(\zeta_{k}\right),y\left(\zeta_{k}\right),z\left(\zeta_{k}\right)\right)d{\mathbb{P}}_{x,y,z}\geq k{\mathbb{P}}_{x,y,z}\left\{\zeta_{k}\leq t\right\},$$

${\mathbb{P}}_{x,y,z}\left\{\zeta_{k}\leq t\right\}\leq \frac{V(x,y,z)\exp\left\{K_{1}t\right\}}{k}$ for all *k* ≥ 1 and hence

$${\mathbb{P}}_{x,y,z}\left\{\zeta_{k}>t\right\}\geq 1-\frac{V(x,y,z)\exp\left\{K_{1}t\right\}}{k}$$

for all *k* ≥ 1. On the other hand, since *ζ*_*k*_ > *t* implies *V*(*x*(*s*), *y*(*s*), *z*(*s*)) < *k* for all *s* ∈ [0, *t*],

$${\mathbb{P}}_{x,y,z}\{V(x(s),y(s),z(s))<k\forall s\in [0,t]\}\geq 1-\frac{V(x,y,z)\exp\left\{K_{1}t\right\}}{k}$$

for all *k* ≥ 1. Letting *k* → ∞ yields

$${\mathbb{P}}_{x,y,z}\{V(x(s),y(s),z(s))<\infty \forall s\in [0,t]\}=1.$$

As *V*(*x*(*s*), *y*(*s*), *z*(*s*)) < ∞ implies 1 − *x*(*s*) > 0, so ${\mathbb{P}}_{x,y,z}\{0<x(s)<1\forall s\in [0,t]\}=1$. Since *t* > 0 is arbitrary,${\mathbb{P}}_{x,y,z}\{0<x(s)<1\forall s\geq 0\}=1$.

We complete the proof. □

##### Proof of Lemma 3.2.

By Ito’s formula, since 0 ≤ *x*(*t*) ≤ 1 for all *t* ≥ 0 a.s. (by Lemma 3.1),

$$d(x(t)z(t))=x(t)dz(t)+z(t)dx(t)+dx(t)dz(t)\;\;\;\;\;\;\;\;\;\;\;\;\;\;\;\;\;\;\;\;\;\;\;=\left[bx(t)+\left(1+\tau_{2}^{2}-ax(t)-c\right)x(t)z(t)-ax^{2}(t)z^{2}(t)\right]dt\;\;\;\;\;\;\;\;\;\;\;\;\;\;\;\;\;\;\;\;\;\;\;\;\;\;\;\;\;\;-\tau_{2}x(t)z(t)dW_{2}+\tau_{3}x(t)z(t)dW_{3}\;\;\;\;\;\;\;\;\;\;\;\;\;\;\;\;\;\;\;\;\;\;\;+\left[rx(t)z(t)(1-x(t)-y(t))-ax(t)y(t)z^{2}(t)\right]dt+\tau_{1}x(t)z(t)(1-x(t)-y(t))dW_{1}\;\;\;\;\;\;\;\;\;\;\;\;\;\;\;\;\;\;\;\;\;\;\leq \left[b+\left(1+\tau_{2}^{2}+r\right)x(t)z(t)-ax^{2}(t)z^{2}(t)\right]dt\;\;\;\;\;\;\;\;\;\;\;\;\;\;\;\;\;\;\;\;\;\;\;\;\;+\tau_{1}x(t)z(t)(1-x(t)-y(t))dW_{1}-\tau_{2}x(t)z(t)dW_{2}+\tau_{3}x(t)z(t)dW_{3}.$$

Taking expectation both sides yields

$$\frac{d}{dt}E_{u}x(t)z(t)\leq b+\left(1+\tau_{2}^{2}+r\right)E_{u}x(t)z(t)-aE_{u}x^{2}(t)z^{2}(t)\;\;\;\;\;\;\;\;\;\;\;\;\;\;\;\;\;\;\;\;\;\;\;\;\;\;\;\;\;\;\;\leq b+\left(1+\tau_{2}^{2}+r\right)E_{u}x(t)z(t)-a{\left(E_{u}x(t)z(t)\right)}^{2},$$

here we have used the equality $E_{u}x^{2}(t)z^{2}(t)\geq {\left(E_{u}x(t)z(t)\right)}^{2}$. This implies that

$$\frac{d}{dt}E_{u}x(t)z(t)\leq b+\frac{{\left(1+\tau_{2}^{2}+r\right)}^{2}}{4a}-a{\left(E_{u}x(t)z(t)-\frac{1+\tau_{2}^{2}+r}{2a}\right)}^{2}.$$

Therefore for all *t* ≥ 0 we obtain

$$E_{u}x(t)z(t)\leq \min\left\{1,\frac{b}{a}+{\left(\frac{1+\tau_{2}^{2}+r}{2a}\right)}^{2}\right\}.$$

So, Lemma 3.2 is proved. □

### 3.3. Proofs of Propositions

#### Proof of Proposition 2.1.

$\mu_{1}=\delta_{0}^{*}\times \delta_{0}^{*}\times \pi_{1}$, where the invariant measure *π*_1_ has the inverse gamma distribution with parameters $\alpha =\frac{2\left(c-1-\tau_{2}^{2}\right)}{\tau_{2}^{2}+\tau_{3}^{2}}+1$ and $\beta =\frac{2b}{\tau_{2}^{2}+\tau_{3}^{2}}$. The mean is given by $\frac{\beta }{\alpha -1}=\frac{b}{c-1-\tau_{2}^{2}}$. It is clearly that when *τ*_2_ approaches zero, the mean approaches $\frac{b}{c-1}$ which is the third coordinate of the equilibrium *E*_1_.

$\mu_{2}=\delta_{1}^{*}\times \delta_{0}^{*}\times \pi_{2}$, where the invariant measure *π*_2_ has the generalized inverse Gaussian distribution with parameters $\theta =\frac{2\left(1+\tau_{2}^{2}-a-c\right)}{\tau_{2}^{2}+\tau_{3}^{2}}-1$, $\psi =\frac{4a}{\tau_{2}^{2}+\tau_{3}^{2}}$, and $\chi =\frac{4b}{\tau_{2}^{2}+\tau_{3}^{2}}$. From A.17 on page 172 in [26], we know the mean of this distribution *π*_2_ is $R_{\theta }(w)\sqrt{\frac{b}{a}}$, where $w:=\sqrt{\psi \chi }=\frac{4\sqrt{ab}}{\tau_{2}^{2}+\tau_{3}^{2}}$, $R_{\theta }(w):=\frac{K_{\theta +1}(w)}{K_{\theta }(w)}$ and *K*_*θ*_(*w*) is the modified Bessel function of the third kind with index *θ*.

Since $\frac{\theta }{w}=\frac{2\left(1+\tau_{3}^{2}-a-c\right)}{4\sqrt{ab}}-\frac{\tau_{2}^{2}+\tau_{3}^{2}}{4\sqrt{ab}}$,$\lim_{\left(\tau_{2},\tau_{3}\right)\to (0,0)}\frac{\theta }{w}=\frac{1-a-c}{2\sqrt{ab}}$. From the reference [26], we get

$$R_{\theta }(w)=\frac{\theta }{w}+\sqrt{{\left(\frac{\theta }{w}\right)}^{2}+D_{\theta }(w)},\;\text{where}\;D_{\theta }(w):=\frac{K_{\theta +1}(w)K_{\theta -1}(w)}{K_{\theta }^{2}(w)}.$$

Due to the asymptotic expansion of *D*_*θ*_(*w*) as *w* → ∞ (see A.22 on page 173 in [26])

$$D_{\theta }(w)=1+\frac{1}{w}+\frac{-256\theta^{2}+64}{{\left(8w\right)}^{3}}+o\left(w^{-4}\right)\;(w\to \infty ),$$

it is clear that *D*_*θ*_(*w*) approaches 1 as (*τ*_2_, *τ*_3_) approaches (0,0). Hence

$$\underset{\left(\tau_{2},\tau_{3}\right)\to (0,0)}{\lim}R_{\theta }(w)\sqrt{\frac{b}{a}}=\underset{\left(\tau_{2},\tau_{3}\right)\to (0,0)}{\lim}\left(\frac{\theta }{w}+\sqrt{{\left(\frac{\theta }{w}\right)}^{2}+D_{\theta }(w)}\right)\sqrt{\frac{b}{a}}=\frac{1-a-c+\sqrt{{\left(1-a-c\right)}^{2}+4ab}}{2a}.$$

#### Proof of Proposition 2.3.

We first show the threshold $\lambda =\sqrt{ab}\;R_{\theta }(w)-1-\frac{\tau_{2}^{2}}{2}$ is an increasing function of the virus burst size *b*. Let $h(b)=\sqrt{ab}\;R_{\theta }(w)$ where $w=\frac{4\sqrt{ab}}{\tau_{2}^{2}+\tau_{3}^{2}}$. Since $h^{\prime }(b)=\frac{2a}{\tau_{2}^{2}+\tau_{3}^{2}}\left[R_{\theta }^{2}(w)-2\frac{\theta }{w}R_{\theta }(w)-1\right]$, *h*′ (*b*) ≥ 0 is equivalent to $R_{\theta }(w)\geq \frac{\theta }{w}+\sqrt{{\left(\frac{\theta }{w}\right)}^{2}+1}$. By the integral representation of *D*_*θ*_(*w*) (see A.29 on page 176 in [26]), we have

$$D_{\theta }(w)=1+\frac{2}{w^{2}}\int_{w}^{+\infty }\tilde{w}{\left[\frac{K_{\theta }(\tilde{w})}{K_{\theta }(w)}\right]}^{2}d\tilde{w}\geq 1\;(w>0).$$

It follows that

$$R_{\theta }(w)=\frac{\theta }{w}+\sqrt{{\left(\frac{\theta }{w}\right)}^{2}+D_{\theta }(w)}\geq \frac{\theta }{w}+\sqrt{{\left(\frac{\theta }{w}\right)}^{2}+1}.$$

Therefore *h*(*b*) is increasing w.r.t. *b*.

From the proof of Proposition 2.1,$\lim_{\left(\tau_{2},\tau_{3}\right)\to (0,0)}\lambda =\lim_{\left(\tau_{2},\tau_{3}\right)\to (0,0)}\sqrt{ab}R_{\theta }(w)-1-\frac{1}{2}\tau_{2}^{2}=\frac{1-a-c+\sqrt{{\left(1-a-c\right)}^{2}}+4ab}{2}-1=\bar{\lambda }$. It is easy to see that $\bar{\lambda }=0$ gives $b=1+\frac{c}{a}$ which is the threshold $b_{s_{1}}$ for the deterministic system (1.1). □

## 4. Numerical simulation and discussion

### 4.1. Numerical simulation with biological explanation

This section is devoted to demonstrate our main analytical results in Section 2. Data values from our previous research (see [27] and [12]) are used to estimate parameter values and simulate our stochastic model. The maximal radius of the tumor in mice brain, which is considered to be dead from the tumor, is 5 millimeters. Because our SDE model neglects spatial variations, tumor size is converted into cell numbers using the constant of cell density *K* = 10^6^ per cubical millimeter. After non-dimensionalization, the parameter values are *r* = 0.36, *a* = 0.11, and *c* = 0.44. For simplicity, we carry out numerical simulations based on the non-dimensionalized SDE systems (2.2) and (2.3). The quantities *x*, *y*, and *v* are, respectively, the portion of uninfected tumor cells, infected tumor cells, and free virus particles over the maximal cell density of the tumor *C*. These quantities are not absolute numbers but relative numbers. We just call them relative uninfected tumor cells and so on in the figures below. Notice that the quantity *z* in the system (2.3) is the ratio of relative free virus particles over relative infected tumor cells. In our simulation, time is scaled or relative time since *T* = *δt*. In [27] and [12], the parameter *b*, the burst size of free virus particles, plays a pivotal role in determining the success of glioma virotherapy. So we will simulate the trajectories of the system (2.2) with the initial value (0.5, 0.5, 1.5) and the modified system (2.3) with the initial value (0.5, 0.5, 3) as *b* and noise intensities are varied while all the other parameters are fixed.

#### Example 1.

**Figure 1.**
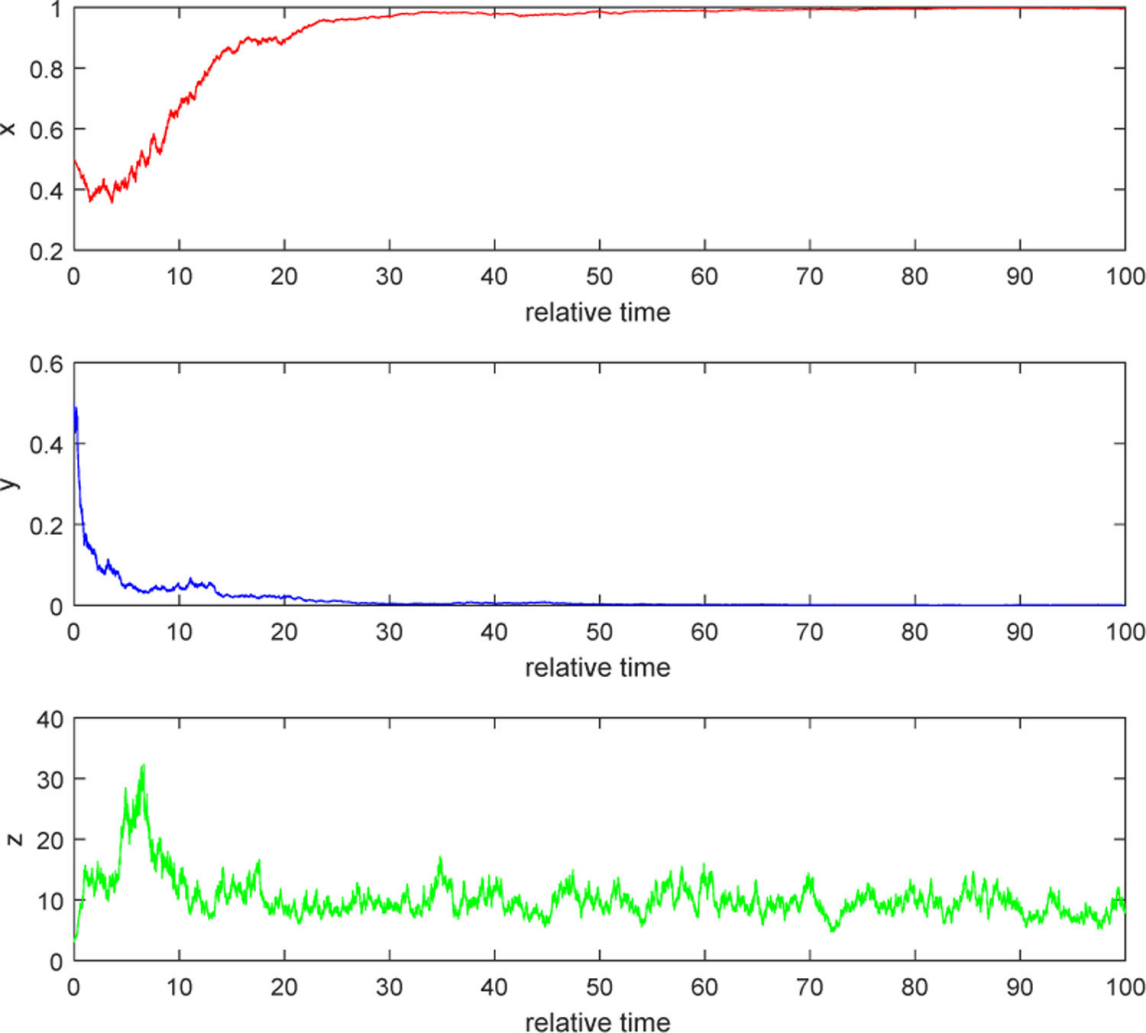
Stochastic solution paths of (2.3) when *b* = 5, *τ*_1_ = 0.2, *τ*_2_ = 0.3, and *τ*_3_ = 0.2

**Figure 2.**
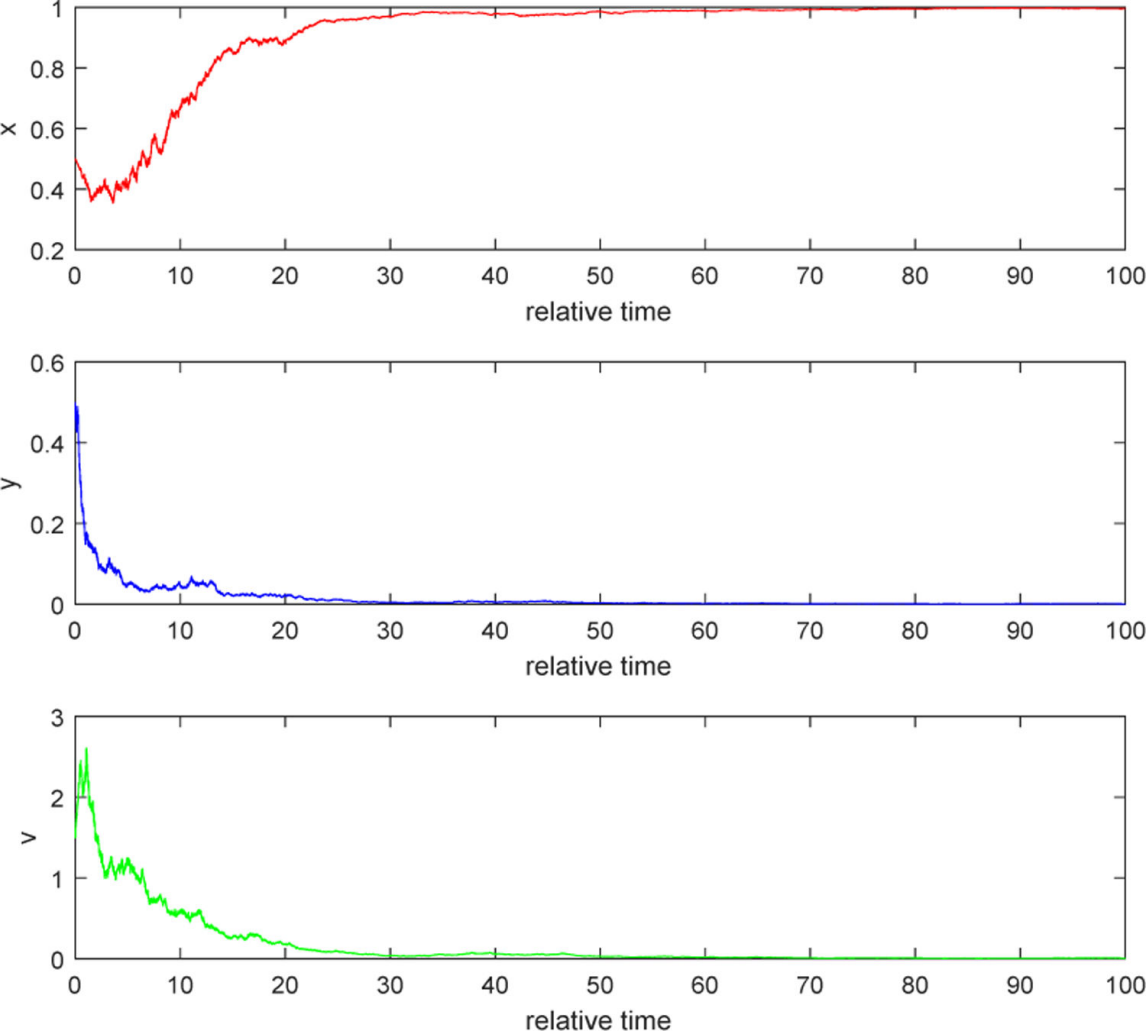
Stochastic solution paths of (2.2) when *b* = 5, *τ*_1_ = 0.2, *τ*_2_ = 0.3, and *τ*_3_ = 0.2

We illustrate the situation when *λ* < 0. In the figures 1 and 2, we take *b* = 5, *τ*_1_ = 0.2, *τ*_2_ = 0.3, and *τ*_3_ = 0.2. By computation, *θ* = 7.3077, *w* = 22.8191, *R*_*θ*_(*w*) = 1.39, and hence *λ* = −0.0141 < 0. Figure 1 indicates that the relative uninfected tumor cells increase to the relative maximal cell density of the tumor, which is 1; the relative infected tumor cells decay to zero; and the ratio of relative free virus particle over relative infected tumor cells reaches an equilibrium state, which explains why the relative free viruses are wiped out as in Figure 2. These two pictures verify the conclusion in Case 1 of Theorem 2.2. In terms of biological meaning, since the burst size *b* is not big enough, the number of new viruses released from a lysis of an infected cell is inconsiderable when compared with the number of free viruses dying out. Because of that, in the early stage, the population of free virus particles increases by contribution from lysis of some infected tumor cells but later the number of free viruses decrease and decay to zero. The decrease in free viruses leads to decrease in infected tumor cells and hence the infected also decay to zero. Then the uninfected becomes less and less infected by free viruses, and finally increases to its carrying capacity. Therefore, virotherapy fails.

#### Example 2.

**Figure 3.**
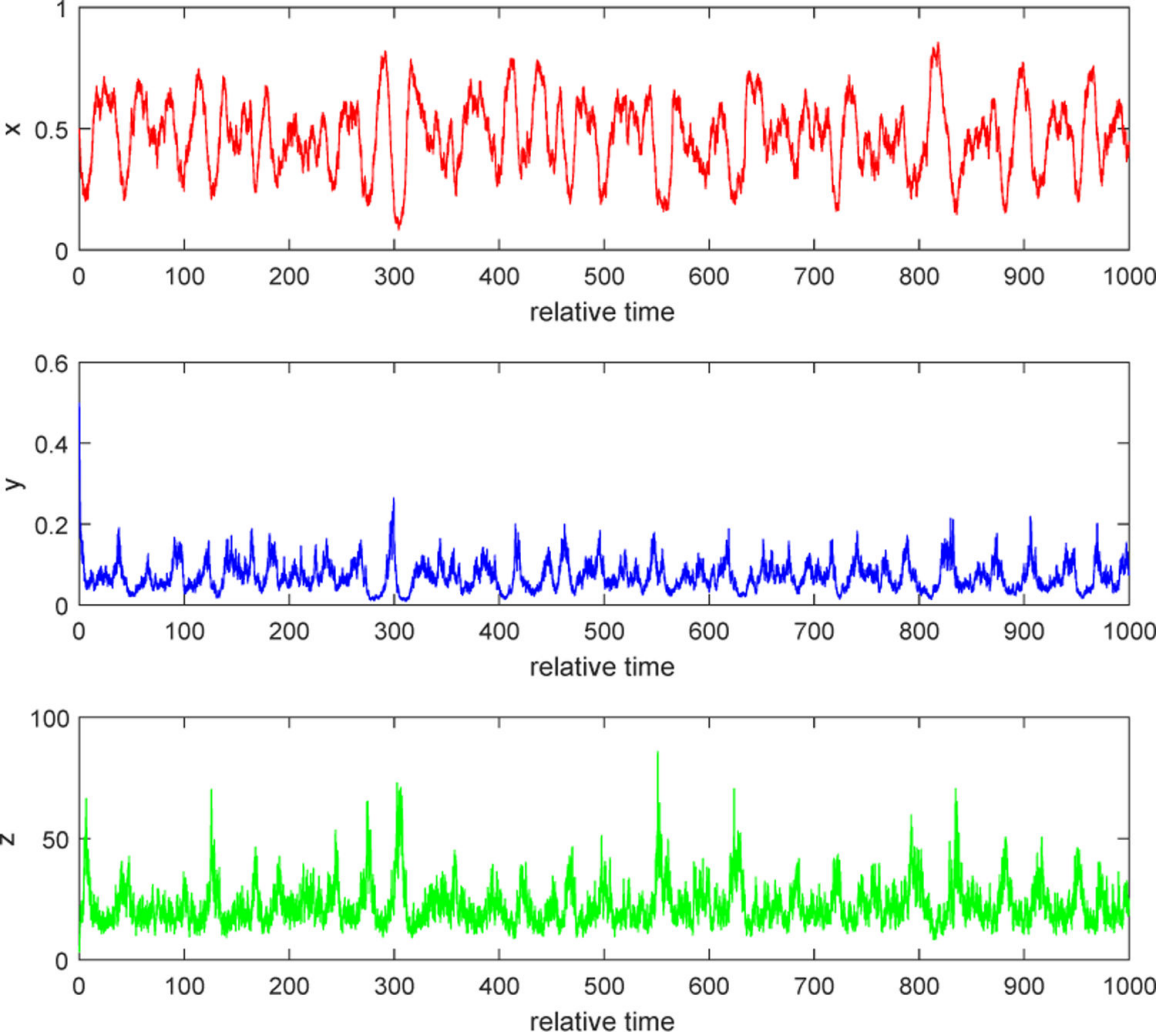
Stochastic solution paths of (2.3) when *b* = 10, *τ*_1_ = 0.2, *τ*_2_ = 0.3, and *τ*_3_ = 0.2

**Figure 4.**
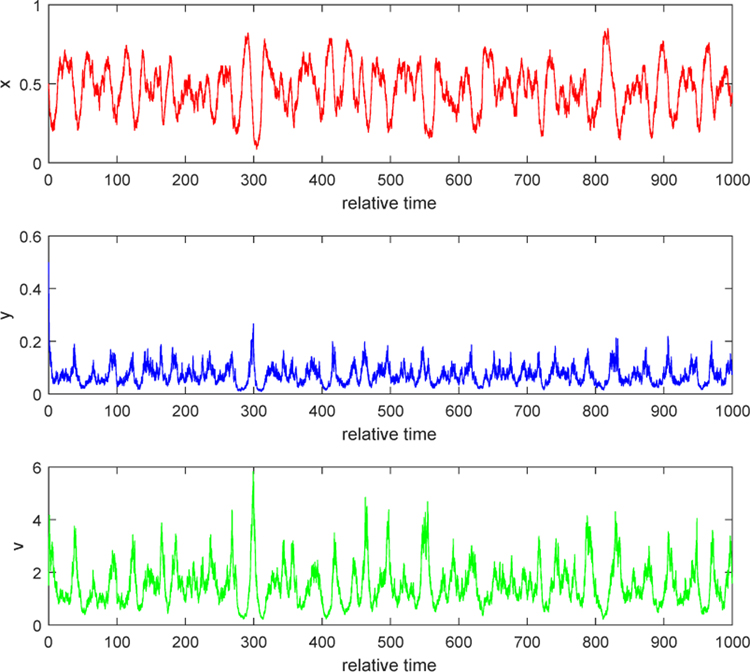
Stochastic solution paths of (2.2) when *b* = 10, *τ*_1_ = 0.2, *τ*_2_ = 0.3, and *τ*_3_ = 0.2

We consider the situation when *λ* > 0. Take *b* = 10 and noise intensities have the same values as in Example 1. By computation, we can obtain *λ* = 0.2832 > 0. The second conclusion in Case 1 of Theorem 2.2 indicates that relative populations of uninfected, infected tumor cells and free viruses persist strongly and finally settle down into a coexistence equilibrium state. Both Figures 3 and 4 show that, as time goes by, each solution path oscillates most of the time around a positive equilibrium point (which is the positive equilibrium point of the corresponding ODE system, *E*_3_ or *Q*_3_). Biologically, this phenomenon can be explained as follows. When the burst size of free viruses is increased, say, to 10, the number of new viruses from a lysis of an infected tumor cell becomes significant. Then, the dying-out infected cells contribute much to the number of free viruses within the tumor. The number of free virus particles is big enough to prevent the growth of uninfected tumor cells. Some of the uninfected getting infected by free viruses becomes the infected, while some of them keep growing. Three populations interact in the mutually coexistent way. This shows that injecting free viruses with stronger burst size into the tumor yields better treatment.

**Figure 5.**
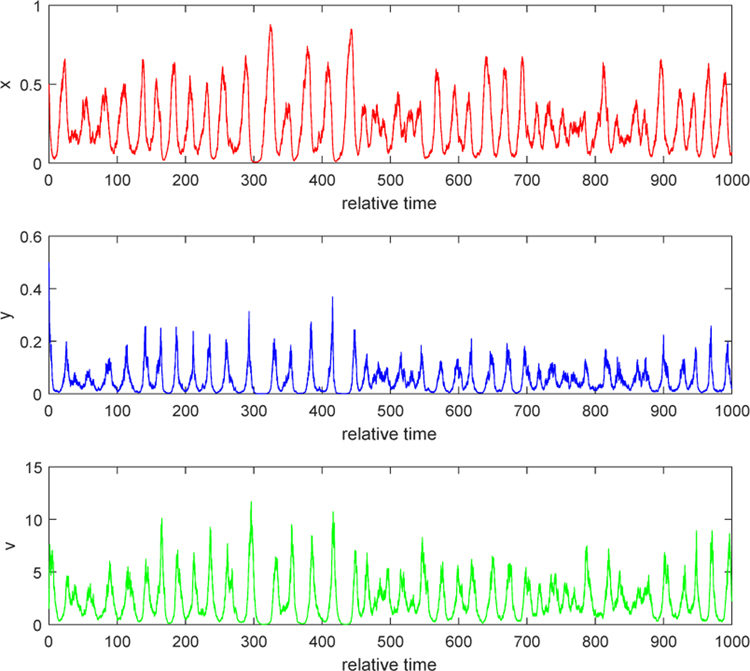
Stochastic solution paths of (2.2) when *b* = 20, *τ*_1_ = 0.2, *τ*_2_ = 0.3, and *τ*_3_ = 0.2

**Figure 6.**
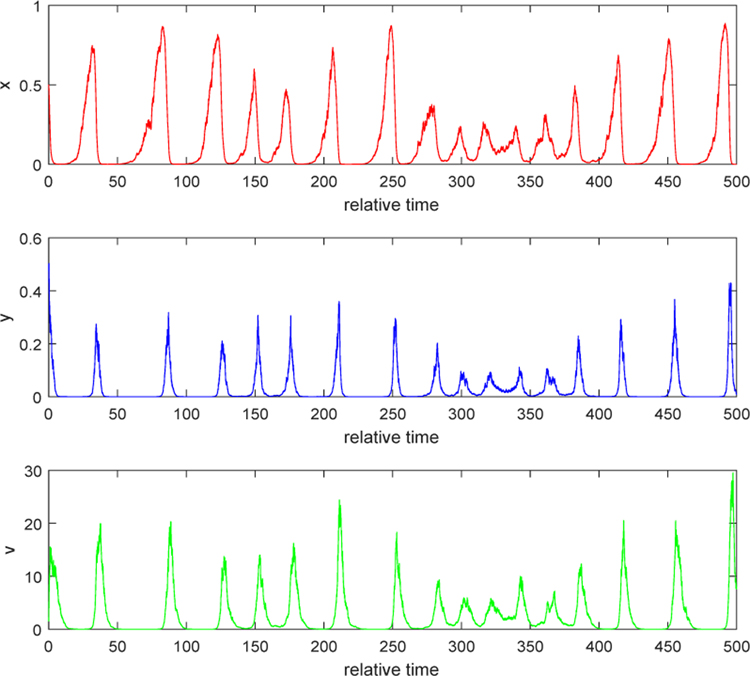
Stochastic solution paths of (2.2) when *b* = 40, *τ*_1_ = 0.2, *τ*_2_ = 0.3, and *τ*_3_ = 0.2

**Figure 7.**
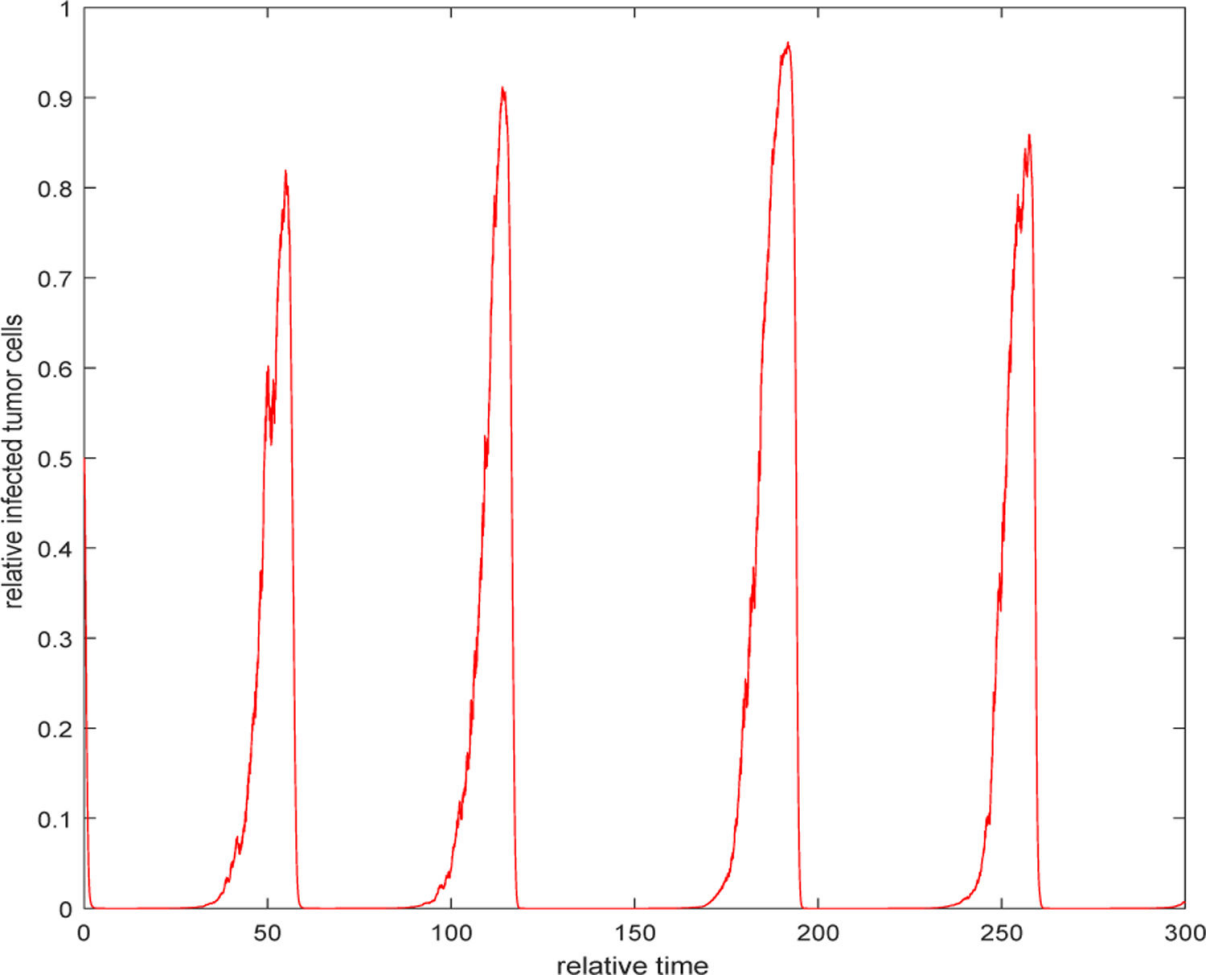
The relative uninfected tumor cells when *b* = 80, *τ*_1_ = 0.2, *τ*_2_ = 0.3, and *τ*_3_ = 0.2

If the burst size *b* is doubled to 20 while the noise intensities are the same as in Example 1 and 2, Figure 5 indicates that all solution paths still persist and oscillate most of the time around an equilibrium state. The difference is that the tumor load, which is the total number of the uninfected and the infected cells, is much smaller than the tumor load with the burst size *b* = 10. When we increase the burst size *b* to 40 and noise intensities are kept the same, the solution behaves differently. Figure 6 shows that all solution paths represent a pulsating oscillatory. The minimum of the uninfected tumor population can reach a very small value comparing with the maximum tumor size. In this case, the tumor may be regarded to be undetectable and then we consider that the tumor is eradicated. This phenomenon becomes more visible when the burst size is taken very large, say *b* = 80, as illustrated in Figure 7. Thus, the viral treatment can be seen to be some success.

### 4.2. Discussion

In this paper, our basic virotherapy model of stochastic type is able to predict the dynamics of viral therapy based on the viral burst size *b* and noise intensities. We found thresholds *ζ* and *λ* that provide conditions for various outcomes of our stochastic model. The parameter *ζ* combines infected tumor cell lysis rate *δ*, virus degradation rate *γ*, and their stochastic variation *τ*_2_ and *τ*_3_, which describes possibilities if the tumor can be eradicated by viral therapy. The parameter *λ* is a differential function of the viral burst size *b* and the noise intensities *τ*_2_ and *τ*_3_, and is increasing as *b* increases. We elaborate some medical implications of these parameters and medical outcomes theoretically in Section 2. We also numerically demonstrate these dynamical outcomes and present more biological explanations in Subsection 4.1. We compare our stochastic model and its deterministic counterpart. Equilibrium states of deterministic models correspond to ergodic invariant probability measures of stochastic models. However, our stochastic model demonstrate some new features. For the deterministic model, there is no possibility to eradicate the tumor, but for the stochastic model, there is a case where the tumor can be eradicated. This is due to introducing a big variance $\tau_{1}^{2}$ of tumor cell growth rate.

There are several interesting questions arisen in our study. For two ergodic invariant probability measures on the boundary, we obtain their explicit probability distributions, so that we can compute their expectations which correspond equilibrium solutions of the deterministic system. For the ergodic invariant probability measure supported by the interior of the domain, we are unable to find its probability distribution explicitly in this study although we know it correspond the coexistence equilibrium solution of the deterministic model. One question is to find the explicit expression of this probability distribution. A second question is about Hopf bifurcations. In the deterministic model, when the viral burst size passes through the second threshold value $b_{s_{2}}$, there is a Hopf bifurcation with appearance of three families of periodic solutions. We know that *λ* is an increasing function of the viral burst size *b*, and its threshold $b_{s_{1}}$ also serve well for classification of solutions to stochastic model. We ask if there is a Hopf bifurcation for the stochastic model when the viral burst size *b* passes through $b_{s_{2}}$. This would be very interesting from both theoretical and practical viewpoint.

One of the major challenges in current medical practice of oncolytic viral therapy is to get insight into the complexity of the immune responses. Understanding the dynamics of oncolytic virotherapy in the presence of immune responses is a considerable need. The innate immune response has a tendency to reduce the efficacy of oncolytic viral treatment by lowering new virus multiplication and blocking the infection spreading, while the stimulated adaptive immune response tends to reduce tumor cells. So the extension of our stochastic model to incorporate the innate and adaptive immune systems is expected. We plan to conduct these studies in the future.

## Appendix.

We state some properties of the Generalized Inverse Gaussian Distribution GIG(*θ*, *χ*, *ψ*) without proof (see [26] for more details), that is needed in Section 3, where θ∈ℝ, *χ* > 0, and *ψ* > 0. Its probability density function is given by

$$f(z;\theta ,\chi ,\psi )=\frac{{\left(\psi /\chi \right)}^{\theta /2}}{2K_{\theta }(\sqrt{\chi \psi })}z^{\theta -1}\exp\left\{-\frac{1}{2}\left(\chi z^{-1}+\psi z\right)\right\},z\in (0,\infty ),$$

where *K*_*θ*_(·) is the modified Bessel function of the third kind with index *θ*, given by

$$K_{\theta }(\phi )=\frac{1}{2}\int_{0}^{\infty }x^{\theta -1}\exp\left\{-\frac{1}{2}\phi \left(x+\frac{1}{x}\right)\right\}dx,\phi >0.$$

Let $w=\sqrt{\chi \psi }$ and $\eta =\sqrt{\frac{\chi }{\psi }}$, then the above probability density function takes the form

$$f(z;\theta ,w,\eta )=\frac{\eta^{-\theta }}{2K_{\theta }(w)}z^{\theta -1}\exp\left\{-\frac{1}{2}w\left(\frac{\eta }{z}+\frac{z}{\eta }\right)\right\},z>0.$$

Moments of random variable *X* ~ GIG(*θ*, *w*, η) are given by

$$EX^{p}=\frac{K_{\theta +p}(w)}{K_{\theta }(w)}\eta^{p},\;p\geq 0.$$

## References

[1] Cross D, Burmester J, Gene therapy for cancer treatment: past, present, and future, Clinical Medicine & Research, 4 (2006), 218–227. 16988102 10.3121/cmr.4.3.218 PMC1570487

[2] Anguela XM, High KA, Entering the modern era of gene therapy, Annu. Rev. Med., 70 (2019), 273–288. 30477394 10.1146/annurev-med-012017-043332

[3] Chiocca EA, Oncolytic viruses, Nature Reviews Cancer, 2 (2002), 938–950. 12459732 10.1038/nrc948

[4] Kelly E and Russell SJ, History of oncolytic viruses: genesis to genetic engineering, Molecular Therapy, 15 (2007), 651–659. 17299401 10.1038/sj.mt.6300108

[5] Andtbacka RHI, Kaufman HL, Collichio F , Talimogene laherparepvec improves durable response rate in patients with advanced melanoma, Journal of Clinical Oncology, 33 (2015), 2780–2788. 26014293 10.1200/JCO.2014.58.3377

[6] Liu T and Kirn D, Gene therapy progress and prospects cancer: oncolytic viruses, Gene Therapy, 7 (2008), 2–8. 10.1038/gt.2008.72 18418413

[7] Wodarz D, Viruses as antitumor weapons: defining conditions for tumor remission, Cancer Research, 61 (2001), 3501–3507. 11309314

[8] Wodarz D, Gene therapy for killing p53-negative cancer cells: use of replicating versus nonreplicating agents, Human Gene Therapy, 159 (2003), 153–159. 10.1089/104303403321070847 12614566

[9] Dingli D, Cascino MD, Josic K, Russell SJ, and Bajzer Z, Mathematical modeling of cancer radiovirotherapy, Mathematical Biosciences, 199 (2006), 55–78. 16376950 10.1016/j.mbs.2005.11.001

[10] Bajzer Z, Carr T, Josic K, Russell SJ, and Dingli D, Modeling of cancer virotherapy with recombinant measles viruses, Journal of Theoretical Biology, 252 (2008), 109–22. 18316099 10.1016/j.jtbi.2008.01.016

[11] Komarova NL and Wodarz D, ODE models for oncolytic virus dynamics, Journal of Theoretical Biology, 263 (2010), 530–543. 20085772 10.1016/j.jtbi.2010.01.009 PMC2839021

[12] Tian JP, The replicability of oncolytic virus: defining conditions on tumor virotherapy, Mathematical Biosciences and Engineering, 8 (2011), 841–860. 21675814 10.3934/mbe.2011.8.841

[13] Phan TA, Tian JP, The Role of the Innate Immune System in Oncolytic Virotherapy, Computational and Mathematical Methods in Medicine, Volume 2017, Article ID 6587258, 17 pages. 10.1155/2017/6587258 PMC5742943 29379572

[14] Yuan Y, Allen LJ, Stochastic models for virus and immune system dynamics, Mathematical Biosciences, 234 (2011), 84–94. 21945381 10.1016/j.mbs.2011.08.007 PMC3249397

[15] Kim KS, Kim S, and Jung IH, Dynamics of tumor virotherapy: A deterministic and stochastic model approach, Stochastic Analysis and Applications, 34 (2016), 483–495.

[16] Rajalakshmi M and Ghosh M, Modeling treatment of cancer using virotherapy with generalized logistic growth of tumor cells, Stochastic Analysis and Applications, 36 (2018), 1068–1086.

[17] Rajalakshmi M and Ghosh M, Modeling treatment of cancer using oncolytic virotherapy with saturated incidence, Stochastic Analysis and Applications, 38 (2020), 565–579.

[18] Allen E, Modeling with Ito Stochastic Differential Equations, Springer, Dordrecht, The Netherlands, 2007.

[19] Cresson J, Puig B, and Sonner S, Validating stochastic models: invariance criteria for systems of stochastic differential equations and the selection of a stochastic Hodgkin-Huxley type model, Int. J. Biomath. Biostat., 2 (2013), 111–122.

[20] Cresson J, Puig B, and Sonner S, Stochastic models in biology and the invariance problem, Discrete and Continuous Dynamical Systems Series B, 21 (2016), 2145–2168.

[21] Cresson J and Sonner S, A note on a derivation method for SDE models: applications in biology and viability criteria, Stochastic Analysis and Applications, 36 (2018), 224–239.

[22] Phan TA, Tian JP, and Wang B, Dynamics of cholera epidemic models in fluctuating environments, Stochastics and Dynamics, 2020. Available from: https://www.worldscientific.com/doi/pdf/10.1142/S0219493721500118. 10.1142/s0219493721500118 PMC8881056 35221416

[23] Hening A and Nguyen HD, Coexistence and extinction for stochastic Kolmogorov systems, Ann. Appl. Probab, 28 (2018), 1893–1942.

[24] Mao X, Stochastic differential equations and applications, 2^*nd*^ edition, Woodhead Publishing Limited, 2007.

[25] Ikeda N, Watanabe S, Stochastic Differential Equations and Diffusion Processes, 2^*nd*^ edition, North-Holland Publishing Co., Amsterdam, 1989.

[26] Jorgensen B, Statistical Property of the Generalized Inverse Gaussian Distribution, Springer-Verlag New York, 1982.

[27] Friedman A, Tian JP, Fulci G, Chiocca EA, and Wang J, Glioma virotherapy: the effects of innate immune suppression and increased viral replication capacity, Cancer Research, 66 (2006), 2314–2319. 16489036 10.1158/0008-5472.CAN-05-2661

